# Factors contributing to spatial–temporal variations of observed oxygen concentration over the Qinghai-Tibetan Plateau

**DOI:** 10.1038/s41598-021-96741-6

**Published:** 2021-08-30

**Authors:** Peijun Shi, Yanqiang Chen, Gangfeng Zhang, Haiping Tang, Zhi Chen, Deyong Yu, Jing Yang, Tao Ye, Jing’ai Wang, Shunlin Liang, Yonggui Ma, Jidong Wu, Peng Gong

**Affiliations:** 1 State Key Laboratory of Surface Processes and Resource Ecology, Beijing Normal University, Beijing, 100875 China; 2 Academy of Plateau Science and Sustainability, People’s Government of Qinghai Province and Beijing Normal University, Xining, 810016 China; 3 Academy of Disaster Reduction and Emergency Management, Ministry of Emergency Management and Ministry of Education, Beijing Normal University, Beijing, 100875 China; 4 Faculty of Geographical Science, Beijing Normal University, Beijing, 100875 China; 5 College of Geographical Science, Qinghai Normal University, Xining, 810016 China; 6 Department of Geographical Sciences, University of Maryland, College Park, MD 20742 USA; 7 College of Life Science, Qinghai Normal University, Xining, 810016 China; 8 Ministry of Education Key Laboratory of Earth System Modeling, Department of Earth System Science, Tsinghua University, Beijing, 100084 China

**Keywords:** Cryospheric science, Climate-change impacts, Natural hazards

## Abstract

Oxygen (O_2_) is the most abundant molecule in the atmosphere after nitrogen. Previous studies have documented that oxygen concentration remains nearly constant (20.946%) at all altitudes. Here we show for the first time that oxygen concentration varies significantly from earlier consensus and shows strong spatial and seasonal differences. Field observations on the Qinghai-Tibetan Plateau (QTP) indicate oxygen concentration of 19.94–20.66% (2018, n = 80), 19.98–20.78% (2019, *n* = 166) and 19.97–20.73% (2020, n = 176), all statistically different from earlier reports (*p* < 0.001) and are lower than the nearly constant. The mean oxygen concentration in summer (20.47%) is 0.31% higher than that of winter (20.16%) (n = 53) at identical locations in 2019, sampled in the Qilian Mountains, northwest QTP. We used LMG (The Lindeman, Merenda and Gold) method to estimate the relative contributions of altitude, air temperature and vegetation index (Fractional Vegetation Cover, FVC and Leaf Area Index, LAI) to oxygen concentration, which are 47%, 32% and 3% (FVC, R^2^ = 82%); 45%, 30% and 7% (LAI, R^2^ = 82%), respectively. These findings provide a new perspective for in-depth understanding on population risk in high altitude regions in the context of global climate change, to ensure the health and safety of residents and tourists in high altitude regions and promoting the stability, prosperity and sustainable development of high-altitude regions worldwide.

## Introduction

The Qinghai-Tibetan Plateau lies between 26°00′–39°47′N, 73°19′–104°47′E, covers an area of ~ 2.5 × 10^6^ km^2^, with an average altitude exceeding 4500 m. It stretches from the southern Himalayas to the northern Kunlun Mountains and Qilian Mountains, with a total length of 1500 km, while it spans about 2900 km from the Hindu Kush Mountains and western Pamir Plateau, to the eastern Hengduan Mountains and Yunnan-Guizhou Plateau^1^. Also known as the “Roof of the World”, the climate on the QTP is characterized by a typical cold and dry alpine climate, with an average annual temperature from − 3.1 to 4.4 °C and an average annual precipitation from 103 to 694 mm, which both decreases from east to west^2–4^. The environmental condition of the QTP, especially the western part, is quite harsh because of high altitude, intense radiation and thin air, but it is vital for soil and water conservation, carbon sequestration and biodiversity conservation of China and even Asia^5^.

One of the key features that enable our planet Earth to house an active and diverse biology is the presence of free molecular oxygen (O_2_) in the atmosphere. In the long history of Geological time scale, near-surface oxygen concentration has changed drastically and induced critical impacts on the biogeochemical processes of the earth surface, and the evolution of life. However, for the past century, oxygen concentration has been regarded as nearly constant^6–9^ since the first result presented by Benedict (1912)^10^. Machta and Hughes^11^ collected observations of atmospheric oxygen between 50°N and 60°S, mainly over the oceans, and suggested an almost constant O_2_ value of 20.946% by volume in dry air^11^. Studies further suggest that oxygen concentration remain nearly constant, with little variations at different altitudes and within different seasons^12–15^.

**Figure 1:**
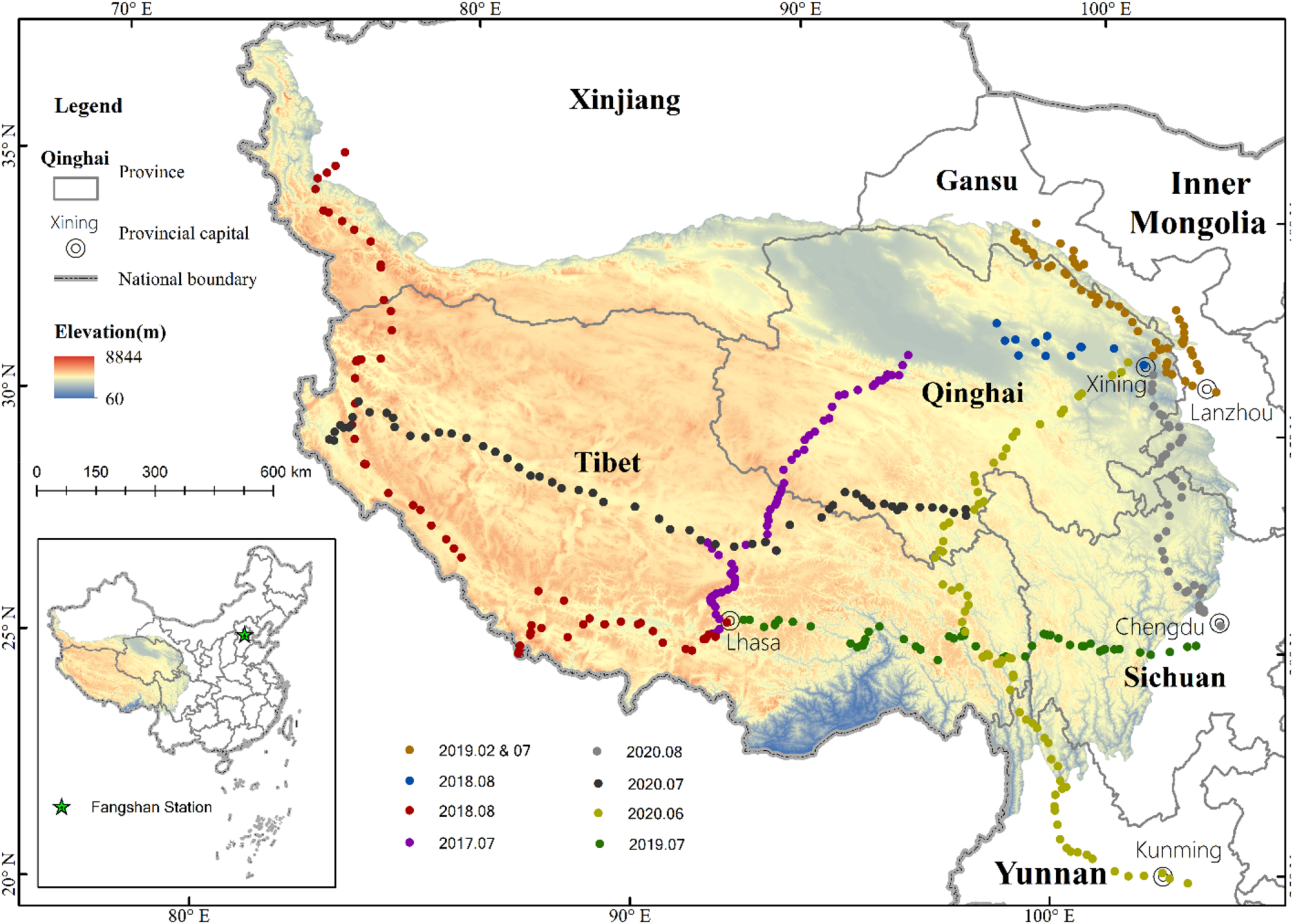
Location of the sampled sites (lower left: the QTP and Fangshan Station in China) (prepared by YC in ArcMap 10.3, https://www.esri.com/zh-cn/arcgis/products/arcgis-pro/resources).

Here, for the first time, we systematically investigate the spatial–temporal variation of near-surface oxygen concentration and quantify the relative contributions of altitude, temperature, and vegetation to it. We conduct field observations on the QTP and its vicinities and collect 487 samples, with altitude varied from 645 to 5238 m above sea level. On each site, information collected included location, altitude, oxygen concentration, barometric pressure and air temperature. As the control group, a fixed-site continuous observation is conducted in Fangshan, Beijing with an altitude of 33 m (Fig. 1).

## Results

### Variations of oxygen concentration over the QTP

**Figure 2:**
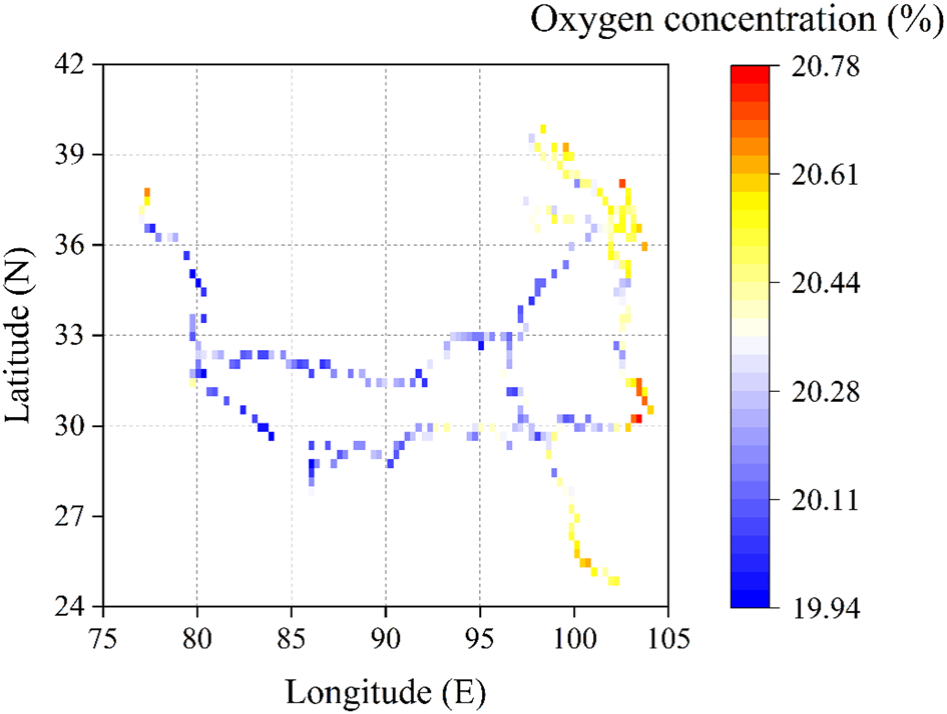
Spatial distribution of oxygen concentration within the QTP and its neighboring regions in the summers of 2018–2020.

**Figure 3:**
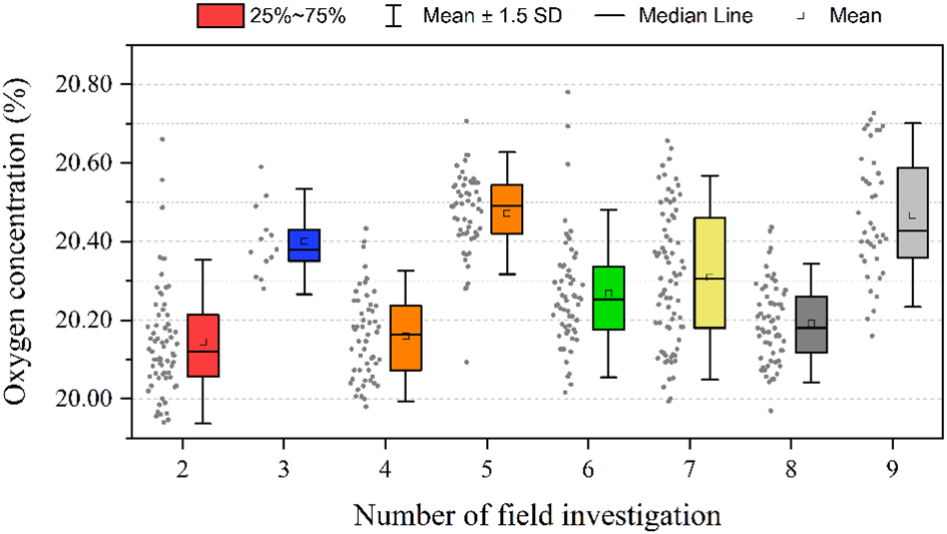
Box plots of oxygen concentration of field investigations during 2018–2020. The numbers of field investigation are the same with those in Table S2 of Supplementary Materials.

Our results indicate significant difference of oxygen concentrations from the constant assumed (20.946%)^11^. In our sample by electrochemical oxygen meter, oxygen concentrations range from 19.94 to 20.78% and are lower than the nearly constant (Table 1). The oxygen concentrations sampled in the margin areas of QTP (which locates in/near Tarim Basin, Qinghai Lake, Qilian Mountains, and in Hengduan Mountains and Yunnan-Guizhou Plateau are relatively higher, while those in the hinterland of the plateau are relatively lower (Fig. 2, Table S1, Table S2). Moreover, the distribution of oxygen concentration for each field investigation also has high variation (Fig. 3) as the samples were taken along with different investigation routes and landforms over the QTP.

**Table 1:** Statistics of oxygen concentration and altitude of 2018–2020.

Year	Sample size	Factor	Mean	Max	Min	SD	*t* test statistics
2018	80	Oxygen concentration (%)	20.19	20.66	19.94	0.16	*p* < 0.001, 95% CI: 20.15–20.22%
		Altitude (m)	4006	5238	1352	822	
2019	166	Oxygen concentration (%)	20.30	20.78	19.98	0.18	*p* < 0.001, 95% CI: 20.27–20.33%
		Altitude (m)	3000	5026	645	909	
2020	176	Oxygen concentration (%)	20.30	20.73	19.97	0.18	*p* < 0.001, 95% CI: 20.28–20.33%
		Altitude (m)	3562	5362	534	1116

### Decreasing oxygen concentration by altitude

**Figure 4:**
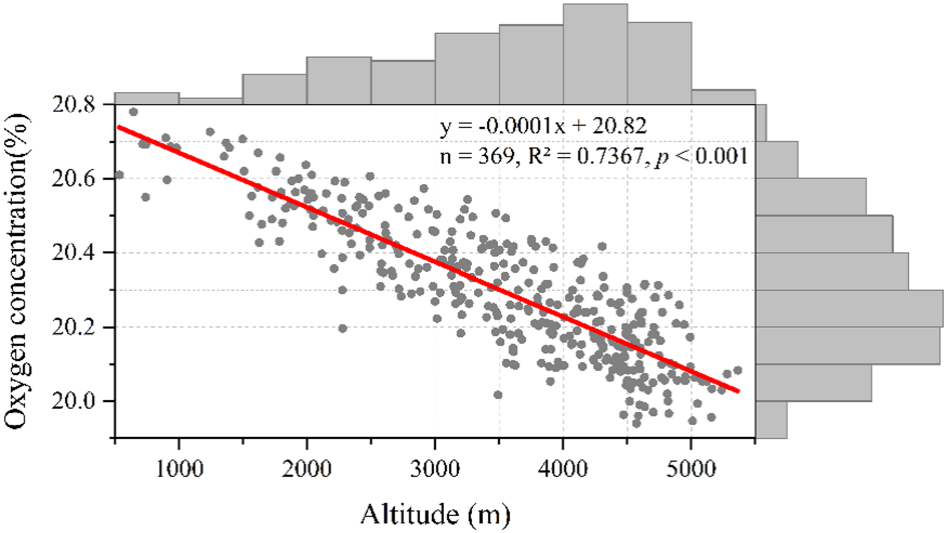
Decreasing oxygen concentration as a function of altitude (winter samples excluded)^16^. The red line indicates the linearly fitted trend of oxygen concentration. The upper and right histograms depict the frequency distribution of altitude and oxygen concentration, respectively.

Oxygen concentration shows pronounced linear relationship with altitude, and the slope is negative and significant (Fig. 4, n = 369, R^2^ = 0.7367, *p* < 0.001). For each 1000 m increase in altitude, oxygen concentration is going to drop by 0.15% in summer.

### Influence on oxygen concentration from air temperature and vegetation

**Figure 5:**
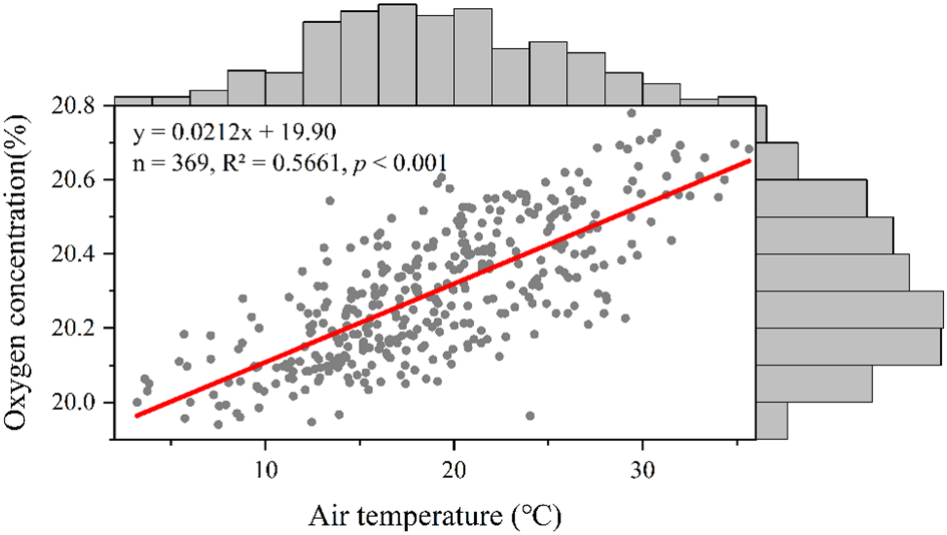
Scatter plot and linear fitting of oxygen concentration and air temperature (winter samples excluded)^16^. The upper and right histograms depict the frequency distribution of air temperature and oxygen concentration, respectively.

Air temperature is directly proportional to oxygen concentration (Fig. 5, n = 369, R^2^ = 0.5661, *p* < 0.001), for all the samples in 2018–2020 (winter samples excluded). There are also strong seasonal variations in oxygen concentration. Data from 53 paired-samples (winter vs. summer), each pair at identical locations in Qilian Mountains, northeast of the QTP, indicate oxygen concentrations in summer (n = 53, mean: 20.47%) are statistically higher than those in winter (n = 53, mean: 20.16%) (Table S3).

A continuous fixed-point observation at Fangshan Station, Beijing indicates strong seasonal and diurnal variation in oxygen concentration (Figure S1). For monthly means, the highest oxygen concentration (21.31%) appeared in June 2019 (Figure S2, Table S4), and the lowest appeared in January 2020 (20.29%), which indicated a month-scale range of 1.02%. The daily variation of oxygen concentration has with a minimum value between 4:00 to 7:00 h (Beijing Time, hereinafter inclusive) (mean: 20.67%) and a maximum value between 13:00 and 15:00 h (mean: 21.14%), with an average diurnal range of 0.47%. Overall, the lowest oxygen concentration (19.69%) appeared at 7:43 on December 31, 2019, and the highest oxygen concentration (21.64%) appeared at 15:28 on June 14, 2019 (Figure S3).

**Figure 6:**
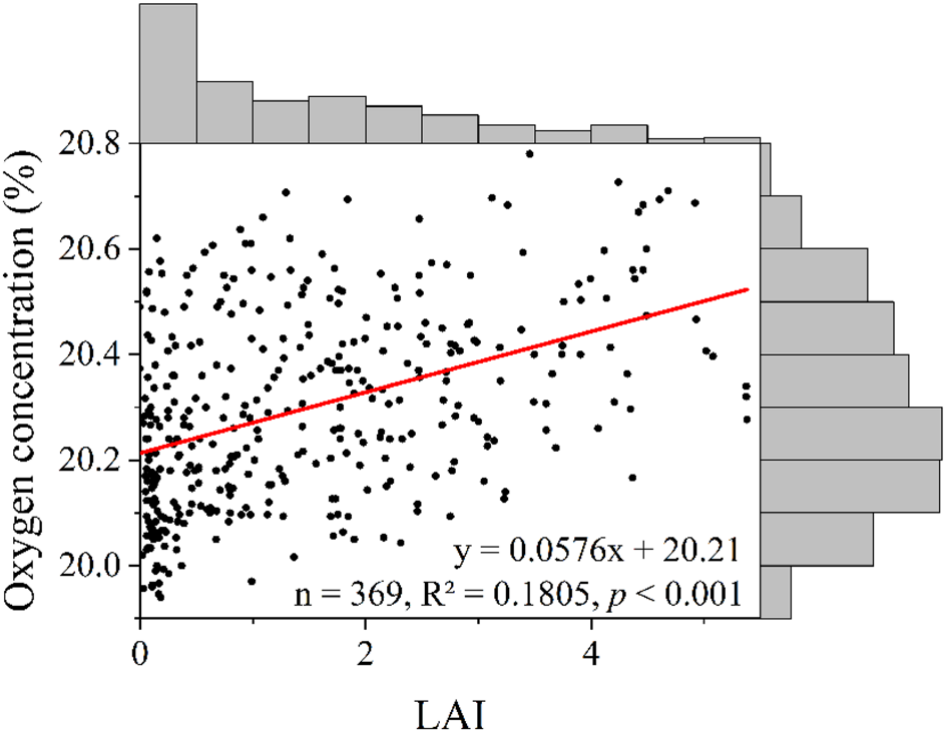
Scatter plot of LAI and oxygen concentration. The upper and right histograms in the right part represent the frequency distribution of LAI and oxygen concentration, respectively.

Oxygen is mainly produced from the photosynthesis of green plants, the process by which plants use sunlight energy to produce organic compounds from CO_2_ and water. The ability of an ecosystem to sequester CO_2_ and produce O_2_ is largely determined by its vegetation conditions^17,18^. During the fieldwork on the QTP, we took 33 survey sample area of 1 km × 1 km to estimate local FVC, which was then compared and examined to be well fitted with the estimated FVC and LAI retrieved from remote sensing data (Table S5, Figures S4 and S5). The FVCs had significantly positive correlations with oxygen concentration (Figure S5). The LAI, which was validated with local FVC, also had a significantly positive correlation with oxygen concentration (Fig. 6 and Fig. S4). There was a distinctive disparity in the FVCs between winter and summer in the Qilian Mountains, which intertwined with the air temperature differences, and resulted in oxygen concentration variations between the two seasons. Due to the low mean air temperature in winter (− 1.96 °C), most vegetation physiological activities stopped and the amount of oxygen production remained at a very low level.

### Factors influencing oxygen concentration and their relative contributions

The LMG method in total explained 82% of the variation in oxygen concentration, among which, altitude contributed to 47%, air temperature 32% and FVC 3% (Table 2). Similarly, the relative contributions of altitude, air temperature and LAI to oxygen concentration are 45%, 30%, and 7%, respectively (R^2^ = 82%). Both calculations are based on all the samples except those in winter of 2019 (n = 369).

**Table 2:** Relative contributions of altitude, air temperature, FVC (LAI) to oxygen concentration in the QTP (unit: %).

Factor	Relative contribution (relative importance)	95% confidence interval		R^2^
Altitude	46.89	43.52	50.12	81.66
Air temperature	31.57	27.52	35.52	
FVC	3.20	1.51	5.37	
Altitude	44.74	40.97	48.39	81.55
Air temperature	29.98	26.05	33.73	
LAI	6.83	4.43	9.80	

## Discussion

### Factors’ relative contributions to oxygen concentration

In the study by Shi et al. (2019), the main factors affecting oxygen concentration on the QTP included altitude, 500 hPa air temperature and FVC. 500 hPa air temperature was negatively correlated with oxygen concentration^19^, but our results show a positive correlation between near-surface air temperature and oxygen concentration. Although the two air temperature datasets came from different sources, their relationships with the oxygen concentration should have presented a certain consistency. What caused such a mismatch can be owed to how well the two datasets represented the local conditions. The 500 hPa air temperature, which was derived from ERA-Interim reanalysis temperature data (the European Centre for Medium-Range Weather Forecasts) and has a spatial resolution of 0.25° × 0.25° (roughly 30 km × 30 km), may introduce some errors when applied directly to the sample sites due to its relatively coarse spatial and temporal resolutions^20^. The real-time measured air temperature used in this study should be much more reliable, and the controlled investigations in Qilian Mountains during winter and summer and the observations at Fangshan Station, Beijing further confirmed this positive correlation.

### O_2_ concentration over the QTP under global climate change

The QTP has been experiencing unprecedented warming under global climate change, largely intensified by human activities^5^. Meteorological records revealed that the warming rate on the QTP was twice of that observed globally over the past five decades^21^, although some studies suggested that the warming hiatus is also occurred over the QTP^22^, induced by internal to decadal climate variations originating from the Pacific and Atlantic. This warming trend is projected to continue in the future, based on the outputs from both global climate models and regional climate models^23–25^. Evidence gleaned from satellite observations, long-term ecological stations, and modeling works have all consistently shown that the QTP ecosystems have been greening under climate warming—with a clear north–south disparity^26,27^. To date, the most important limiting factor on the most part of this region for vegetation growth is temperature^28,29^. Vegetation productivity firstly increases and then decreases with temperature rises (the temperature corresponding to the maximum productivity is called optimal temperature, T_opt_). Chen et al. (2021) found that a remarkable geographical heterogeneity in T_opt_ was observed over the QTP^30^. Higher T_opt_ values generally appeared in the north-eastern parts, while the south-western QTP had relatively lower T_opt_ (< 10 °C), and the average T_opt_ of non-forest vegetation on the QTP was ~ 14.7 °C. Moreover, the QTP was a source of atmospheric O_2_ from 2000 to 2013, although the O_2_ consumption showed an increasing trend over the QTP in recent decades^31,32^. Thus, enhanced vegetation growth under a warming climate before a certain threshold will greatly improve the capacity of oxygen production of these ecosystems. A warming climate and favorable vegetation growth conditions on the QTP over the past decades could have led to rising oxygen concentration. While it will weaken the vegetation growth if the air temperature exceeds a certain threshold in the future, even though great uncertainties in climate projections still exist.

From a global perspective, much more attention should be paid to the population risks of permanent residents and short-lived visitors to high altitude regions (≥ 2500 m above sea level), which cover a total area of ~ 11 million km^2^, accounting for 7.7% of the Earth’s land surface. In 2015, the total population in these areas was ~ 107 million, accounting for 1.5% of the world's total population^33^. It has been estimated that more than 100 million tourists travel to high-altitude regions by the year 2000^34^, and this number keeps increasing in many parts of these regions such as the QTP^16^, Indian Himalayan Region^35^, and the European Alps^36^. Therefore, we suggest the science community and stakeholders pay more attention to this overlooked issue and investigate the spatial and temporal heterogeneity of O_2_ by sampled and fixed continuous observations. More studies with fixed continuous observations and meteorological, remote sensing, reanalysis, statistical, and clinical data of hypoxia-related fields, will further validate and consolidate these findings at a much more accurate spatial and temporal scale. These works will promote multi- and cross-disciplinary studies on geoscience and high-altitude medicine to improve the health of at-risk populations and ensure the sustainable development of high-altitude regions.

## Data and methods

From 2017 to 2020, we conducted several field investigations on the QTP and its vicinities. We collected 487 samples located within 76.9336–104.0639 E and 24.8350–39.7990 N, with altitude varied from 645 to 5238 m above sea level (Fig. 1, Tables S1 and S2). On each site, we record geographical location, altitude, barometric pressure, air temperature and oxygen concentration (by electrochemical oxygen meter). For all sampled sites, each of these parameters was simultaneously measured with three instruments and then averaged for later analysis. 33 fractional vegetation covers (FVCs) were also measured in the field with 1 km × 1 km survey sample area^37^ then the ones for other sample sites were interpolated by FVC_GLASS since the linear fit coefficient was the highest (Figure S4) among FVC and the retrieved FVCs (FVC_GE, FVC_MODIS and FVC_GLASS) (Table S5). Continuous in situ observation is carried out at Fangshan Station, Beijing (39.6914 N and 116.0547 E, altitude: 33 m, Fig. 1) as the control group data, where oxygen concentration is automatically recorded by electrochemical oxygen meter for every ~ 2 min. The boundary data of the QTP is provided by the Resource Environmental Science and Data Center, Institute of Geographical Sciences and Natural Resources Research, Chinese Academy of Sciences (http://www.resdc.cn).

We use altitude (Alt), near-surface temperature (Tem) and fractional vegetation cover (FVC, or Leaf Area Index, LAI) to explain the variation of oxygen concentration via linear regression:

1 $${\text{Oxygen concentration }} = b_{0} + b_{1} \times {\text{Alt }} + b_{2} \times {\text{Tem}} + b_{3} \times {\text{FVC }} ( {{\text{LAI}}} ) + {\varepsilon }$$

where ε is the random error representing factors that are not considered but might contribute to variations in oxygen concentration^38^. To further determine the relative importance of variables, we apply the method developed by Lindemann, Merenda and Gold (LMG)^39^. The LMG method calculates the relative contribution of each variable to the R^2^ with the consideration of the sequence of predictors appearing in the model. The relative contributions of the factors (altitude (Alt), air temperature (Tem), FVC/LAI) to oxygen concentration are estimated using a bootstrap technique based on the LMG method.

## Supplementary Information

Supplementary Information.

## Data Availability

Data used in this study are available from the corresponding author upon request.

## References

[1] Zhang Y, Li B, Zheng D (2002). A discussion on the boundary and area of the Tibetan Plateau in China. Geogr. Res..

[2] Frauenfeld OW, Zhang TJ, Serreze MC (2005). Climate change and variability using European Centre for Medium-Range Weather Forecasts reanalysis (ERA-40) temperatures on the Tibetan Plateau. J. Geophys. Res..

[3] Ding J, Yang T, Zhao Y (2018). Increasingly important role of atmospheric aridity on Tibetan alpine grasslands. Geophys. Res. Lett..

[4] Wang X, Pang G, Yang M (2018). Precipitation over the Tibetan Plateau during recent decades: A review based on observations and simulations. Int. J. Climatol..

[5] Yao T, Xue Y, Chen D (2019). Recent Third Pole’s rapid warming accompanies cryospheric melt and water cycle intensification and interactions between monsoon and environment: Multi-disciplinary approach with observation, modeling and analysis. Bull. Am. Meteorol. Soc..

[6] Krogh, A. The composition of the atmosphere. *Det Kgl. Darzske Vidertskabernes Selskab. ***1**, 1 (1919).

[7] Shepherd, M. *The composition of the atmosphere at approximately 21.5 kilometers, U.S. Army Stratosphere Flight of 1935 in Balloon Explorer II*. National Geographic Society, 117–133 (1935).

[8] Carpenter TM (1937). The constancy of the atmosphere with respect to carbon dioxide and oxygen content. J. Am. Chem. Soc..

[9] Lockhart EE, Court A (1942). Oxygen deficiency in Antarctic air. Mon. Weather Rev..

[10] Benedict FG (1912). The Composition of the Atmosphere with Special Reference to its Oxygen Content.

[11] Machta L, Hughes E (1970). Atmospheric oxygen in 1967 to 1970. Science.

[12] Keeling RF, Shertz SR (1992). Seasonal and interannual variations in atmospheric oxygen and implications for the global carbon cycle. Nature.

[13] Cynthia MB (2003). High-altitude adaptations. Lancet.

[14] Cynthia MB (2007). Two routes to functional adaptation: Tibetan and Andean high-altitude natives. Proc. Natl. Acad. Sci. U.S.A..

[15] Stephens BB, Long MC, Keeling RF (2018). The O_2_/N_2_ ratio and CO_2_ Airborne Southern Ocean (ORCAS) study. Bull. Am. Meteorol. Soc..

[16] Shi P, Chen Y, Ma H (2021). Further research on the factors contributing to oxygen concentration over the Qinghai-Tibetan Plateau. Chin. Sci. Bull..

[17] Zhang Y, Xiao X, Wu X (2017). A global moderate resolution dataset of gross primary production of vegetation for 2000–2016. Sci. Data.

[18] Zhang L, Zhang B, Li W (2018). Spatiotemporal changes and drivers of global land vegetation oxygen production between 2001 and 2010. Ecol. Ind..

[19] Shi P, Chen Y, Zhang A (2019). Factors contribution to oxygen concentration in Qinghai-Tibetan Plateau. Chin. Sci. Bull..

[20] Zhao P, Gao L, Wei J (2020). Evaluation of ERA-Interim air temperature data over the Qilian Mountains of China. Adv. Meteorol..

[21] Chen D, Xu B, Yao T (2015). Assessment of past, present and future environmental changes on the Tibetan Plateau. Chin. Sci. Bull..

[22] Cai D, You Q, Fraedrich K (2017). Spatiotemporal temperature variability over the Tibetan Plateau: Altitudinal dependence associated with the global warming Hiatus. J. Clim..

[23] IPCC (2013). The Physical Science Basis: Contribution of Working Group I to the Fifth Assessment Report of the Intergovernmental Panel on Climate Change.

[24] Su F, Duan X, Chen D (2013). Evaluation of the global climate models in the CMIP5 over the Tibetan plateau. J. Clim..

[25] Zhu X, Wang W, Fraedrich K (2013). Future climate in the Tibetan plateau from a statistical regional climate model. J. Clim..

[26] Piao S, Tan K, Nan H (2012). Impacts of climate and CO_2_ changes on the vegetation growth and carbon balance of Qinghai-Tibetan grasslands over the past five decades. Glob. Planet. Change.

[27] Shen M, Piao S, Chen X (2016). Strong impacts of daily minimum temperature on the green-up date and summer greenness of the Tibetan Plateau. Glob. Change Biol..

[28] Cai D, Fraedrich K, Sielmann F (2015). Vegetation dynamics on the Tibetan Plateau (1982–2006): An attribution by ecohydrological diagnostics. J. Clim..

[29] Wang C, Meng T, Zhai P (2021). Change in drought conditions and its impacts on vegetation growth over the Tibetan plateau. Adv. Clim. Change Res..

[30] Chen A, Huang L, Liu Q (2021). Optimal temperature of vegetation productivity and its linkage with climate and elevation on the Tibetan plateau. Glob. Change Biol..

[31] Liu X, Huang J, Huang J (2019). Impact of anthropogenic activities on global land oxygen flux. Earth. Syst. Sci. Data Discuss..

[32] Liu X, Huang J, Huang J (2020). Estimation of gridded atmospheric oxygen consumption from 1975 to 2018. J. Meteorol. Res..

[33] Zhang A, Wang J, Jiang Y (2018). Spatiotemporal changes of hazard intensity-adjusted population exposure to multiple hazards in Tibet during 1982–2015. Int. J. Disaster Risk Sci..

[34] Burtscher M, Bachmann O, Hatzl T (2001). Cardiopulmonary and metabolic responses in healthy elderly humans during a 1-week hiking programme at high altitude. Eur. J. Appl. Physiol..

[35] Singh, V. & Kotru, R. *Sustainable Tourism in the Indian Himalayan Region. Report of Working Group II*. NITI Aayog (2018). http://niti.gov.in (Accessed 7 July 2021).

[36] Bausch T, Gartner WC (2020). Winter tourism in the European Alps: Is a new paradigm needed?. J. Outdoor Recreat. Tour..

[37] Lin D, Gao Y, Wu Y (2017). A conversion method to determine the regional vegetation cover factor from standard plots based on large sample theory and TM images: A case study in the eastern farming-pasture ecotone of Northern China. Remote Sens..

[38] Zhang Z, Wang L (2017). Advanced Statistics Using R.

[39] Grömping U (2006). Relative importance for linear regression in R: The package relaimpo. J. Stat. Softw..

