# Supplementary material for: Factors contributing to spatial–temporal variations of observed oxygen concentration over the Qinghai-Tibetan Plateau
Source: Sci Rep. 2021 Aug 30;11:17338. doi: 10.1038/s41598-021-96741-6 (PMC8405649; doi:10.1038/s41598-021-96741-6)
Supplement: Supplementary file 1 — Supplementary Information. [file 41598_2021_96741_MOESM1_ESM.doc]

**Supplementary materials for “Factors contributing to spatial-temporal variations of observed oxygen concentration over the Qinghai-Tibetan Plateau”**

Peijun Shi^1, 2, 3, 4, 5*^ Yanqiang Chen^1, 4^ Gangfeng Zhang^1,3,4^ Haiping Tang^1, 4^ Zhi Chen^2, 6^ Deyong Yu^1, 4^ Jing Yang^1, 3, 4^ Tao Ye^3, 1^ Jing’ai Wang^4, 1^ Shunlin Liang^6^ Yonggui Ma^7^ Jidong Wu^1, 3, 4^ Peng Gong^8^

1 State Key Laboratory of Surface Processes and Resource Ecology, Beijing Normal University, Beijing 100875, China

2 Academy of Plateau Science and Sustainability, People’s Government of Qinghai Province and Beijing Normal University, Xining 810016, China

3 Academy of Disaster Reduction and Emergency Management, Ministry of Emergency Management and Ministry of Education, Beijing Normal University, Beijing 100875, China

4 Faculty of Geographical Science, Beijing Normal University, Beijing 100875, China

5 College of Geographical Science, Qinghai Normal University, Xining 810016, China

6 Department of Geographical Sciences, University of Maryland, College Park, MD 20742, USA

7 College of Life Science, Qinghai Normal University, Xining 810016, China

8 Ministry of Education Key Laboratory of Earth System Modeling, Department of Earth System Science, Tsinghua University, Beijing 100084, China

* Correspondence and requests for materials should be addressed to Peijun Shi (spj@bnu.edu.cn)

Table of content

Tables S1 to S5

Figures S1 to S5

References S1

**Table S1**. Data obtained on the QTP in 2017-2020

| ID_year | ID | Time | Longitude (°E) | Latitude (°N) | Altitude (m) | Air temperature (℃) | Barometric pressure (hPa) | Oxygen concentration (%) |
| --- | --- | --- | --- | --- | --- | --- | --- | --- |
| 17-001 | QZ-001 | 2017/7/28 8:59 | 91.1221 | 29.6536 | 3668 |  | 651 | 21.5 |
| 17-002 | QZ-002 | 2017/7/28 14:36 | 90.8613 | 29.3820 | 3596 |  | 653 | 21.4 |
| 17-003 | QZ-003 | 2017/7/28 15:12 | 90.7599 | 29.3426 | 3587 |  | 653 | 20.9 |
| 17-004 | QZ-004 | 2017/7/28 16:11 | 90.9353 | 29.4786 | 3613 |  | 650 | 21.4 |
| 17-005 | QZ-005 | 2017/7/29 9:15 | 90.8788 | 29.7052 | 3725 |  | 646 | 21.9 |
| 17-006 | QZ-006 | 2017/7/29 9:41 | 90.7686 | 29.7924 | 3820 |  | 638 | 21.6 |
| 17-007 | QZ-007 | 2017/7/29 10:05 | 90.7195 | 29.9616 | 3967 |  | 627 | 21.3 |
| 17-008 | QZ-008 | 2017/7/29 10:22 | 90.6291 | 30.0312 | 4109 |  | 616 | 21.4 |
| 17-009 | QZ-009 | 2017/7/29 10:33 | 90.5796 | 30.0599 | 4219 |  | 607 | 21.4 |
| 17-010 | QZ-010 | 2017/7/29 10:46 | 90.5458 | 30.1110 | 4293 |  | 602 | 21.0 |
| 17-011 | QZ-011 | 2017/7/29 10:56 | 90.5776 | 30.1683 | 4448 |  | 590 | 21.1 |
| 17-012 | QZ-012 | 2017/7/29 11:04 | 90.6161 | 30.1973 | 4516 |  | 585 | 20.9 |
| 17-013 | QZ-013 | 2017/7/29 11:17 | 90.6781 | 30.2666 | 4556 |  | 582 | 21.3 |
| 17-014 | QZ-014 | 2017/7/29 11:30 | 90.8168 | 30.3159 | 4302 |  | 602 | 20.8 |
| 17-015 | QZ-015 | 2017/7/29 11:48 | 90.9654 | 30.4052 | 4219 |  | 608 | 21.4 |
| 17-016 | QZ-016 | 2017/7/29 12:09 | 91.1011 | 30.4818 | 4273 |  | 603 | 21.2 |
| 17-017 | QZ-017 | 2017/7/29 12:35 | 91.1209 | 30.5628 | 4370 |  | 595 | 21.2 |
| 17-018 | QZ-018 | 2017/7/29 12:42 | 91.1148 | 30.5862 | 4506 |  | 585 | 21.0 |
| 17-019 | QZ-019 | 2017/7/29 12:51 | 91.1091 | 30.6146 | 4772 |  | 564 | 20.9 |
| 17-020 | QZ-020 | 2017/7/29 12:58 | 91.1039 | 30.6487 | 4939 |  | 552 | 21.3 |
| 17-021 | QZ-021 | 2017/7/29 13:08 | 91.0975 | 30.6817 | 5125 |  | 540 | 21.2 |
| 17-022 | QZ-022 | 2017/7/29 13:45 | 90.9978 | 30.7513 | 4744 |  | 569 | 21.0 |
| 17-023 | QZ-023 | 2017/7/29 14:42 | 90.9623 | 30.7728 | 4735 |  | 569 | 21.1 |
| 17-024 | QZ-024 | 2017/7/29 15:23 | 91.0355 | 30.8912 | 4730 |  | 569 | 21.4 |
| 17-025 | QZ-025 | 2017/7/29 15:47 | 90.9429 | 30.9968 | 4743 |  | 569 | 21.4 |
| 17-026 | QZ-026 | 2017/7/29 16:38 | 90.5568 | 31.1374 | 4598 |  | 579 | 21.1 |
| 17-027 | QZ-027 | 2017/7/29 17:04 | 90.3510 | 31.2607 | 4649 |  | 575 | 21.1 |
| 17-028 | QZ-028 | 2017/7/29 17:26 | 90.1993 | 31.3755 | 4645 |  | 575 | 21.0 |
| 17-029 | QZ-029 | 2017/7/29 18:45 | 91.2264 | 31.4828 | 4750 |  | 567 | 21.2 |
| 17-030 | QZ-030 | 2017/7/30 8:44 | 92.0532 | 31.4706 | 4531 |  | 586 | 20.8 |
| 17-031 | QZ-031 | 2017/7/30 9:48 | 91.7397 | 31.6256 | 4553 |  | 584 | 21.1 |
| 17-032 | QZ-032 | 2017/7/30 10:17 | 91.7498 | 31.8042 | 4789 |  | 566 | 21.1 |
| 17-033 | QZ-033 | 2017/7/30 11:18 | 91.7064 | 31.9991 | 4684 |  | 573 | 20.9 |
| 17-034 | QZ-034 | 2017/7/30 11:39 | 91.7144 | 32.1238 | 4763 |  | 566 | 21.1 |
| 17-035 | QZ-035 | 2017/7/30 12:03 | 91.6788 | 32.2257 | 4652 |  | 576 | 20.9 |
| 17-036 | QZ-036 | 2017/7/31 9:08 | 91.7098 | 32.3762 | 4753 |  | 566 | 21.0 |
| 17-037 | QZ-037 | 2017/7/31 9:37 | 91.8359 | 32.5086 | 4902 |  | 556 | 21.3 |
| 17-038 | QZ-038 | 2017/7/31 10:15 | 91.8515 | 32.5580 | 5142 |  | 539 | 20.9 |
| 17-039 | QZ-039 | 2017/7/31 10:39 | 91.8616 | 32.6602 | 4995 |  | 550 | 20.8 |
| 17-040 | QZ-040 | 2017/7/31 10:57 | 91.9031 | 32.7801 | 5044 |  | 547 | 21.1 |
| 17-041 | QZ-041 | 2017/7/31 11:37 | 91.9184 | 32.8827 | 5221 |  | 531 | 21.0 |
| 17-042 | QZ-042 | 2017/7/31 12:49 | 91.9602 | 33.0305 | 5060 |  | 546 | 20.8 |
| 17-043 | QZ-043 | 2017/7/31 13:27 | 91.8922 | 33.3335 | 4823 |  | 562 | 21.1 |
| 17-044 | QZ-044 | 2017/7/31 13:55 | 92.0665 | 33.5946 | 4713 |  | 568 | 21.0 |
| 17-045 | QZ-045 | 2017/7/31 14:14 | 92.1809 | 33.7509 | 4658 |  | 572 | 21.2 |
| 17-046 | QZ-046 | 2017/7/31 15:02 | 92.3635 | 33.8672 | 4581 |  | 577 | 21.0 |
| 17-047 | QZ-047 | 2017/7/31 15:25 | 92.3401 | 34.0954 | 4755 |  | 565 | 20.9 |
| 17-048 | QZ-048 | 2017/7/31 19:52 | 92.4419 | 34.2150 | 4541 |  | 581 | 21.0 |
| 17-049 | QZ-049 | 2017/8/1 8:12 | 92.5769 | 34.3195 | 4539 |  | 583 | 21.1 |
| 17-050 | QZ-050 | 2017/8/1 8:55 | 92.7822 | 34.6113 | 4697 |  | 572 | 21.1 |
| 17-051 | QZ-051 | 2017/8/1 9:22 | 92.9169 | 34.6795 | 4935 |  | 555 | 20.8 |
| 17-052 | QZ-052 | 2017/8/1 9:59 | 92.9529 | 34.9565 | 4567 |  | 583 | 20.9 |
| 17-053 | QZ-053 | 2017/8/1 10:40 | 93.0894 | 35.2401 | 4662 |  | 575 | 21.0 |
| 17-054 | QZ-054 | 2017/8/1 11:13 | 93.2483 | 35.2860 | 4580 |  | 582 | 20.8 |
| 17-055 | QZ-055 | 2017/8/1 12:15 | 93.6006 | 35.4323 | 4469 |  | 580 | 20.8 |
| 17-056 | QZ-056 | 2017/8/1 13:32 | 94.0158 | 35.5734 | 4656 |  | 575 | 21.1 |
| 17-057 | QZ-057 | 2017/8/1 13:44 | 94.0660 | 35.6268 | 4744 |  | 568 | 21.1 |
| 17-058 | QZ-058 | 2017/8/1 14:05 | 94.1645 | 35.7259 | 4393 |  | 595 | 20.9 |
| 17-059 | QZ-059 | 2017/8/1 14:15 | 94.2645 | 35.7390 | 4230 |  | 607 | 20.8 |
| 17-060 | QZ-060 | 2017/8/1 14:32 | 94.3419 | 35.7992 | 4007 |  | 624 | 21.1 |
| 17-061 | QZ-061 | 2017/8/1 14:45 | 94.3799 | 35.8863 | 3747 |  | 645 | 20.9 |
| 17-062 | QZ-062 | 2017/8/1 14:57 | 94.5107 | 35.8840 | 3653 |  | 651 | 21.0 |
| 17-063 | QZ-063 | 2017/8/1 15:19 | 94.7164 | 35.9072 | 3462 |  | 666 | 20.9 |
| 17-064 | QZ-064 | 2017/8/1 15:57 | 94.7768 | 36.1486 | 3129 |  | 693 | 21.0 |
| 17-065 | QZ-065 | 2017/8/1 16:30 | 94.9019 | 36.4012 | 2832 |  | 718 | 21.1 |
| 18-001 | XZ-001-1 | 2018/8/1 19:30 | 91.0884 | 29.6585 | 3608 | 16.01 | 651.5 | 20.14 |
| 18-002 | XZ-001-2 | 2018/8/2 11:06 | 91.0884 | 29.6585 | 3609 | 17.41 | 654.7 | 20.10 |
| 18-003 | XZ-001-3 | 2018/8/2 11:17 | 91.0884 | 29.6585 | 3611 | 16.41 | 654.6 | 20.13 |
| 18-004 | XZ-002 | 2018/8/3 9:05 | 90.8661 | 29.2906 | 3556 | 14.58 | 660.6 | 20.10 |
| 18-005 | XZ-003 | 2018/8/3 9:45 | 90.6872 | 29.3187 | 3583 | 17.85 | 659.2 | 20.18 |
| 18-006 | XZ-004 | 2018/8/3 10:20 | 90.6300 | 29.2292 | 4063 | 16.11 | 621.1 | 20.15 |
| 18-007 | XZ-005 | 2018/8/3 11:15 | 90.6162 | 29.1950 | 4769 | 18.52 | 570.8 | 20.06 |
| 18-008 | XZ-006 | 2018/8/3 11:40 | 90.5689 | 29.1877 | 4456 | 18.30 | 595.1 | 20.15 |
| 18-009 | XZ-007 | 2018/8/3 12:48 | 90.3323 | 28.8870 | 4601 | 17.92 | 582.8 | 20.09 |
| 18-010 | XZ-008 | 2018/8/3 13:16 | 90.1644 | 28.8992 | 4953 | 14.38 | 557.4 | 20.07 |
| 18-011 | XZ-009 | 2018/8/3 15:43 | 89.5513 | 28.9358 | 4008 | 29.08 | 625.3 | 20.23 |
| 18-012 | XZ-010 | 2018/8/3 16:32 | 89.2411 | 29.1493 | 3895 | 24.15 | 633.1 | 20.27 |
| 18-013 | XZ-011 | 2018/8/3 17:32 | 88.8884 | 29.2737 | 3843 | 22.69 | 637.3 | 20.24 |
| 18-014 | XZ-012 | 2018/8/4 8:12 | 88.8230 | 29.2312 | 3943 | 12.63 | 632.3 | 20.09 |
| 18-015 | XZ-013 | 2018/8/4 10:20 | 88.3768 | 29.2104 | 3897 | 12.37 | 635.9 | 20.19 |
| 18-016 | XZ-014 | 2018/8/4 11:20 | 87.9799 | 29.0755 | 4507 | 12.44 | 589.0 | 20.19 |
| 18-017 | XZ-015 | 2018/8/4 11:38 | 87.9718 | 29.0722 | 4485 | 15.51 | 591.8 | 20.15 |
| 18-018 | XZ-016 | 2018/8/4 12:51 | 87.4372 | 28.9618 | 5122 | 15.24 | 543.7 | 20.05 |
| 18-019 | XZ-017 | 2018/8/4 15:15 | 87.1159 | 28.6037 | 4296 | 13.78 | 606.3 | 20.17 |
| 18-020 | XZ-018 | 2018/8/4 16:32 | 86.4095 | 28.6690 | 4379 | 16.52 | 598.4 | 20.17 |
| 18-021 | XZ-019 | 2018/8/4 17:43 | 86.1672 | 28.5169 | 5086 | 8.05 | 547.3 | 20.05 |
| 18-022 | XZ-020 | 2018/8/5 8:07 | 85.9783 | 28.1620 | 3625 | 9.61 | 649.5 | 20.10 |
| 18-023 | XZ-021 | 2018/8/5 8:48 | 85.9870 | 28.0406 | 2999 | 13.26 | 710.8 | 20.27 |
| 18-024 | XZ-022 | 2018/8/5 9:22 | 85.9777 | 27.9823 | 2212 | 17.69 | 783.7 | 20.36 |
| 18-025 | XZ-023 | 2018/8/5 9:00 | 86.1633 | 28.4486 | 4518 | 6.01 | 592.0 | 20.00 |
| 18-026 | XZ-024 | 2018/8/5 10:13 | 86.1388 | 28.6769 | 4586 | 8.65 | 587.4 | 19.96 |
| 18-027 | XZ-025 | 2018/8/5 15:08 | 87.5619 | 29.1278 | 4074 | 20.72 | 626.6 | 20.16 |
| 18-028 | XZ-026 | 2018/8/5 17:33 | 86.8103 | 29.3901 | 4767 | 15.63 | 569.4 | 20.10 |
| 18-029 | XZ-027 | 2018/8/5 19:07 | 86.1057 | 29.4673 | 5042 | 12.89 | 550.3 | 20.07 |
| 18-030 | XZ-028 | 2018/8/6 9:16 | 83.9376 | 29.7627 | 4572 | 7.50 | 585.9 | 19.94 |
| 18-031 | XZ-029 | 2018/8/6 9:46 | 83.6840 | 29.9133 | 4637 | 9.65 | 581.1 | 19.99 |
| 18-032 | XZ-030 | 2018/8/6 10:57 | 82.9469 | 30.2766 | 4635 | 11.24 | 581.3 | 20.07 |
| 18-033 | XZ-031 | 2018/8/6 13:05 | 82.3348 | 30.6050 | 4952 | 16.12 | 559.5 | 20.06 |
| 18-034 | XZ-032 | 2018/8/6 16:14 | 80.7732 | 31.1781 | 4478 | 13.88 | 593.8 | 20.12 |
| 18-035 | XZ-033 | 2018/8/6 18:29 | 80.1335 | 31.9502 | 4425 | 18.89 | 594.9 | 20.12 |
| 18-036 | XZ-034 | 2018/8/6 10:04 | 83.4224 | 30.0697 | 4599 | 7.55 | 584.9 | 19.99 |
| 18-037 | XZ-035 | 2018/8/6 11:19 | 82.5537 | 30.5499 | 4824 | 13.91 | 562.3 | 19.97 |
| 18-038 | XZ-036 | 2018/8/6 12:53 | 81.6116 | 30.7064 | 4593 | 13.48 | 584.8 | 20.10 |
| 18-039 | XZ-037 | 2018/8/6 14:06 | 80.8074 | 31.1779 | 4548 | 12.30 | 587.4 | 20.08 |
| 18-040 | XZ-038 | 2018/8/6 17:13 | 80.3196 | 31.6595 | 4472 | 24.03 | 591.5 | 19.96 |
| 18-041 | XZ-039 | 2018/8/6 18:38 | 80.0389 | 32.4359 | 4275 | 18.73 | 606.3 | 20.28 |
| 18-042 | XZ-040 | 2018/8/6 19:21 | 80.0998 | 32.5082 | 4303 | 15.17 | 605.4 | 20.15 |
| 18-043 | XZ-041-1 | 2018/8/7 10:09 | 79.8133 | 32.9896 | 4385 | 11.46 | 600.4 | 20.08 |
| 18-044 | XZ-041-2 | 2018/8/7 10:09 | 79.8125 | 32.9896 | 4387 | 11.82 | 601.3 | 20.11 |
| 18-045 | XZ-042 | 2018/8/7 10:57 | 79.7025 | 33.3668 | 4279 | 19.64 | 608.3 | 20.13 |
| 18-046 | XZ-043 | 2018/8/7 11:20 | 79.8131 | 33.4474 | 4260 | 21.05 | 609.1 | 20.17 |
| 18-047 | XZ-044-1 | 2018/8/7 11:43 | 79.7796 | 33.4398 | 4258 | 20.51 | 608.6 | 20.21 |
| 18-048 | XZ-044-2 | 2018/8/7 11:43 | 79.7792 | 33.4393 | 4258 | 20.83 | 609.0 | 20.21 |
| 18-049 | XZ-045 | 2018/8/7 16:58 | 79.7031 | 33.3900 | 4267 | 23.97 | 606.4 | 20.28 |
| 18-050 | XZ-046 | 2018/8/7 17:41 | 79.7095 | 33.3896 | 4265 | 28.04 | 605.8 | 20.29 |
| 18-051 | XZ-047 | 2018/8/8 7:25 | 79.7270 | 33.3787 | 4273 | 13.85 | 607.9 | 20.09 |
| 18-052 | XZ-048 | 2018/8/8 9:13 | 80.3250 | 33.6096 | 4526 | 12.10 | 588.0 | 20.03 |
| 18-053 | XZ-049 | 2018/8/8 10:40 | 80.3698 | 34.3102 | 5238 | 3.73 | 538.6 | 20.03 |
| 18-054 | XZ-050 | 2018/8/8 11:48 | 80.1779 | 34.7292 | 5156 | 5.72 | 547.1 | 19.96 |
| 18-055 | XZ-051 | 2018/8/8 12:29 | 79.8936 | 34.9244 | 5010 | 12.46 | 557.6 | 19.95 |
| 18-056 | XZ-052 | 2018/8/8 14:48 | 79.5178 | 35.6181 | 4890 | 19.13 | 565.4 | 20.06 |
| 18-057 | XZ-053 | 2018/8/8 15:05 | 79.4931 | 35.6849 | 5160 | 15.46 | 545.5 | 20.03 |
| 18-058 | XZ-054 | 2018/8/8 16:33 | 78.9994 | 36.1181 | 4165 | 26.03 | 619.9 | 20.24 |
| 18-059 | XZ-055 | 2018/8/8 17:15 | 78.4435 | 36.2508 | 3864 | 22.99 | 640.4 | 20.32 |
| 18-060 | XZ-056 | 2018/8/9 9:50 | 78.0324 | 36.3481 | 3650 | 17.66 | 657.2 | 20.18 |
| 18-061 | XZ-057 | 2018/8/9 11:10 | 77.5790 | 36.4308 | 4813 | 7.25 | 563.5 | 20.02 |
| 18-062 | XZ-058 | 2018/8/9 11:46 | 77.4157 | 36.4317 | 3903 | 15.18 | 629.1 | 20.14 |
| 18-063 | XZ-059 | 2018/8/9 13:47 | 76.9819 | 36.8364 | 2869 | 25.59 | 715.8 | 20.36 |
| 18-064 | XZ-060 | 2018/8/9 15:04 | 76.9336 | 37.0894 | 3243 | 20.28 | 688.2 | 20.36 |
| 18-065 | XZ-061 | 2018/8/9 15:44 | 77.1314 | 37.2904 | 2188 | 30.45 | 781.9 | 20.49 |
| 18-066 | XZ-062 | 2018/8/9 17:29 | 77.2993 | 37.5117 | 1796 | 32.51 | 817.1 | 20.56 |
| 18-067 | XZ-063 | 2018/8/9 19:43 | 77.4217 | 37.8817 | 1352 | 33.29 | 857.1 | 20.66 |
| 18-068 | QHL-001 | 2018/8/15 8:35 | 101.2273 | 36.6962 | 2659 | 16.21 | 742.8 | 20.35 |
| 18-069 | QHL-002 | 2018/8/15 9:37 | 100.8533 | 36.9878 | 3209 | 14.54 | 701.7 | 20.28 |
| 18-070 | QHL-003 | 2018/8/15 14:02 | 98.8713 | 37.1783 | 3831 | 17.41 | 643.1 | 20.30 |
| 18-071 | QHL-004 | 2018/8/15 16:16 | 98.5498 | 37.0089 | 3118 | 20.58 | 703.7 | 20.43 |
| 18-072 | QHL-005 | 2018/8/15 17:07 | 97.9563 | 37.0317 | 2936 | 16.41 | 718.0 | 20.36 |
| 18-073 | QHL-006 | 2018/8/15 18:17 | 97.3623 | 37.3730 | 2986 | 15.78 | 714.9 | 20.31 |
| 18-074 | QHL-007 | 2018/8/16 8:58 | 97.6494 | 36.9848 | 3061 | 13.27 | 709.7 | 20.38 |
| 18-075 | QHL-008 | 2018/8/16 10:04 | 98.0849 | 36.6686 | 3139 | 21.27 | 702.6 | 20.41 |
| 18-076 | QHL-009 | 2018/8/16 12:41 | 98.8741 | 36.7102 | 3229 | 18.37 | 696.1 | 20.52 |
| 18-077 | QHL-010 | 2018/8/16 14:54 | 99.6057 | 36.7544 | 3785 | 13.10 | 647.6 | 20.42 |
| 18-078 | QHL-011 | 2018/8/16 16:30 | 99.8699 | 36.9810 | 3196 | 20.17 | 697.1 | 20.49 |
| 18-079 | QHL-012 | 2018/8/16 17:02 | 99.9002 | 36.9787 | 3218 | 20.53 | 695.3 | 20.37 |
| 18-080 | QHL-013 | 2018/8/17 17:00 | 101.7371 | 36.6396 | 2294 | 19.12 | 778.2 | 20.59 |
| 19-001 | QL-001 | 2019/2/14 8:53 | 101.9043 | 36.7092 | 2346 | -4.37 | 767.8 | 20.30 |
| 19-002 | QL-002 | 2019/2/14 9:24 | 101.9946 | 36.8545 | 2588 | -2.50 | 744.8 | 20.15 |
| 19-003 | QL-003 | 2019/2/14 9:58 | 102.1736 | 37.0052 | 3377 | -9.10 | 674.0 | 20.13 |
| 19-004 | QL-004 | 2019/2/14 10:26 | 102.2490 | 37.0291 | 3102 | -1.85 | 696.6 | 20.05 |
| 19-005 | QL-005 | 2019/2/14 11:02 | 102.4038 | 37.0487 | 2379 | -0.83 | 762.6 | 20.16 |
| 19-006 | QL-006 | 2019/2/14 11:58 | 102.0693 | 37.1786 | 2611 | 3.71 | 740.5 | 20.20 |
| 19-007 | QL-007 | 2019/2/14 14:24 | 101.5296 | 37.4193 | 3013 | 8.31 | 701.8 | 20.34 |
| 19-008 | QL-008 | 2019/2/14 15:01 | 101.3558 | 37.6281 | 3254 | 3.07 | 679.9 | 20.26 |
| 19-009 | QL-009 | 2019/2/14 15:40 | 101.1146 | 37.8391 | 3770 | -3.36 | 636.7 | 20.18 |
| 19-010 | QL-010 | 2019/2/14 16:07 | 100.9311 | 37.9853 | 3546 | -2.86 | 655.4 | 20.03 |
| 19-011 | QL-011 | 2019/2/14 16:55 | 100.6606 | 38.0224 | 3130 | 3.36 | 691.1 | 20.09 |
| 19-012 | QL-012 | 2019/2/14 17:35 | 100.3153 | 38.1099 | 2860 | -0.50 | 714.9 | 20.22 |
| 19-013 | QL-013 | 2019/2/14 17:56 | 100.2596 | 38.1707 | 2735 | 0.89 | 726.7 | 20.19 |
| 19-014 | QL-014 | 2019/2/15 9:13 | 100.2395 | 38.0150 | 4150 | -11.58 | 609.7 | 20.01 |
| 19-015 | QL-015 | 2019/2/15 10:22 | 100.0957 | 38.2206 | 2693 | -7.32 | 733.5 | 20.11 |
| 19-016 | QL-016 | 2019/2/15 11:08 | 99.7860 | 38.3062 | 3075 | -4.58 | 698.5 | 20.07 |
| 19-017 | QL-017 | 2019/2/15 11:46 | 99.5447 | 38.4557 | 3292 | -2.64 | 678.9 | 20.07 |
| 19-018 | QL-018 | 2019/2/15 12:44 | 99.2527 | 38.6969 | 3552 | -0.05 | 656.0 | 20.05 |
| 19-019 | QL-019 | 2019/2/15 13:23 | 98.8362 | 38.8336 | 3865 | -0.73 | 629.3 | 20.10 |
| 19-020 | QL-020 | 2019/2/15 13:44 | 98.7453 | 38.7842 | 4138 | -6.42 | 607.4 | 19.98 |
| 19-021 | QL-021 | 2019/2/15 14:17 | 98.4150 | 38.8134 | 3361 | 1.93 | 670.0 | 20.02 |
| 19-022 | QL-022 | 2019/2/15 15:23 | 98.2795 | 39.0411 | 4303 | -2.29 | 593.8 | 20.06 |
| 19-023 | QL-023 | 2019/2/15 16:07 | 98.2503 | 39.0939 | 3561 | 2.96 | 653.2 | 20.15 |
| 19-024 | QL-024 | 2019/2/15 16:46 | 98.0556 | 39.1780 | 2998 | 5.36 | 701.2 | 20.23 |
| 19-025 | QL-025 | 2019/2/15 17:26 | 97.9255 | 39.3110 | 2714 | 1.63 | 726.5 | 20.29 |
| 19-026 | QL-026 | 2019/2/15 18:03 | 97.7082 | 39.3411 | 3609 | -1.76 | 648.8 | 20.11 |
| 19-027 | QL-027 | 2019/2/15 18:24 | 97.6492 | 39.3673 | 4047 | -7.87 | 614.0 | 20.00 |
| 19-028 | QL-028 | 2019/2/15 19:00 | 97.6864 | 39.5242 | 3161 | -7.16 | 688.4 | 20.01 |
| 19-029 | QL-029 | 2019/2/16 8:41 | 98.2693 | 39.7990 | 1617 | -11.54 | 838.4 | 20.43 |
| 19-030 | QL-030 | 2019/2/16 10:03 | 99.0575 | 39.3697 | 1569 | -8.69 | 846.5 | 20.18 |
| 19-031 | QL-031 | 2019/2/16 10:55 | 99.4699 | 39.2240 | 1759 | -6.86 | 826.8 | 20.20 |
| 19-032 | QL-032 | 2019/2/16 11:29 | 99.4697 | 39.0421 | 2491 | -3.21 | 753.5 | 20.04 |
| 19-033 | QL-033 | 2019/2/16 11:57 | 99.5355 | 38.9327 | 2794 | -1.09 | 723.8 | 20.07 |
| 19-034 | QL-034 | 2019/2/16 12:26 | 99.6586 | 38.8457 | 2234 | 1.10 | 775.5 | 20.18 |
| 19-035 | QL-035 | 2019/2/16 12:53 | 99.8077 | 38.9238 | 2025 | -2.12 | 796.5 | 20.24 |
| 19-036 | QL-036 | 2019/2/16 17:47 | 102.6323 | 37.9456 | 1497 | -1.19 | 852.5 | 20.40 |
| 19-037 | QL-037 | 2019/2/17 8:42 | 102.7339 | 37.7337 | 1729 | -5.32 | 830.4 | 20.25 |
| 19-038 | QL-038 | 2019/2/17 9:13 | 102.8738 | 37.5764 | 1909 | -3.92 | 811.4 | 20.17 |
| 19-039 | QL-039 | 2019/2/17 9:45 | 102.8836 | 37.4234 | 2151 | -6.55 | 786.6 | 20.09 |
| 19-040 | QL-040 | 2019/2/17 10:20 | 102.8942 | 37.2909 | 2485 | -4.71 | 753.1 | 20.05 |
| 19-041 | QL-041 | 2019/2/17 10:45 | 102.8305 | 37.2094 | 2980 | -7.35 | 706.3 | 20.03 |
| 19-042 | QL-042 | 2019/2/17 11:03 | 102.8651 | 37.1649 | 2800 | -3.98 | 721.9 | 20.04 |
| 19-043 | QL-043 | 2019/2/17 11:44 | 103.0988 | 37.0141 | 2488 | -0.88 | 749.7 | 20.10 |
| 19-044 | QL-044 | 2019/2/17 12:44 | 103.1579 | 36.9001 | 2322 | -0.79 | 767.6 | 20.18 |
| 19-045 | QL-045 | 2019/2/17 13:33 | 103.2787 | 36.6974 | 2051 | 1.22 | 792.9 | 20.22 |
| 19-046 | QL-046 | 2019/2/17 14:05 | 103.3790 | 36.5470 | 1879 | 2.02 | 809.7 | 20.27 |
| 19-047 | QL-047 | 2019/2/17 15:29 | 103.8567 | 36.0485 | 1507 | 2.32 | 847.9 | 20.39 |
| 19-048 | QL-048 | 2019/2/18 9:14 | 103.1643 | 36.1927 | 1648 | -1.54 | 831.5 | 20.27 |
| 19-049 | QL-049 | 2019/2/18 9:52 | 102.9503 | 36.2942 | 1726 | -0.25 | 823.4 | 20.25 |
| 19-050 | QL-050 | 2019/2/18 10:55 | 102.5681 | 36.4387 | 1897 | 0.74 | 804.9 | 20.31 |
| 19-051 | QL-051 | 2019/2/18 11:27 | 102.4064 | 36.4852 | 2005 | 1.41 | 793.6 | 20.24 |
| 19-052 | QL-052 | 2019/2/18 12:08 | 102.3966 | 36.6343 | 2410 | 1.97 | 754.0 | 20.18 |
| 19-053 | QL-053 | 2019/2/18 12:29 | 102.4169 | 36.7154 | 2838 | 1.77 | 714.1 | 20.13 |
| 19-054 | SQL-001 | 2019/7/15 8:40 | 101.9043 | 36.7092 | 2346 | 16.44 | 763.7 | 20.42 |
| 19-055 | SQL-002 | 2019/7/15 9:40 | 101.9946 | 36.8545 | 2588 | 18.88 | 742.2 | 20.42 |
| 19-056 | SQL-003 | 2019/7/15 10:40 | 102.1736 | 37.0052 | 3377 | 15.24 | 676.3 | 20.44 |
| 19-057 | SQL-004 | 2019/7/15 11:44 | 102.2490 | 37.0291 | 3102 | 18.84 | 698.1 | 20.37 |
| 19-058 | SQL-005 | 2019/7/15 12:40 | 102.4038 | 37.0487 | 2379 | 20.95 | 759.1 | 20.52 |
| 19-059 | SQL-006 | 2019/7/15 13:55 | 102.0693 | 37.1786 | 2611 | 23.80 | 738.7 | 20.56 |
| 19-060 | SQL-007 | 2019/7/15 16:50 | 101.5296 | 37.4193 | 3013 | 20.36 | 704.9 | 20.50 |
| 19-061 | SQL-008 | 2019/7/15 17:40 | 101.3558 | 37.6281 | 3254 | 13.42 | 684.3 | 20.54 |
| 19-062 | SQL-009 | 2019/7/15 18:30 | 101.1146 | 37.8391 | 3770 | 11.95 | 642.9 | 20.35 |
| 19-063 | SQL-010 | 2019/7/15 19:10 | 100.9311 | 37.9853 | 3546 | 17.05 | 660.3 | 20.34 |
| 19-064 | SQL-011 | 2019/7/15 19:45 | 100.6606 | 38.0224 | 3130 | 16.09 | 694.6 | 20.46 |
| 19-065 | SQL-012 | 2019/7/15 20:30 | 100.3153 | 38.1099 | 2860 | 15.96 | 716.7 | 20.45 |
| 19-066 | SQL-013 | 2019/7/16 7:32 | 100.2596 | 38.1707 | 2735 | 11.25 | 729.7 | 20.28 |
| 19-067 | SQL-014 | 2019/7/16 8:25 | 100.2395 | 38.0150 | 4150 | 11.17 | 618.0 | 20.09 |
| 19-068 | SQL-015 | 2019/7/16 9:45 | 100.0957 | 38.2206 | 2693 | 15.97 | 732.6 | 20.42 |
| 19-069 | SQL-016 | 2019/7/16 10:58 | 99.7860 | 38.3062 | 3075 | 23.28 | 700.8 | 20.41 |
| 19-070 | SQL-017 | 2019/7/16 11:55 | 99.5447 | 38.4557 | 3292 | 25.12 | 683.0 | 20.50 |
| 19-071 | SQL-018 | 2019/7/16 13:05 | 99.2527 | 38.6969 | 3552 | 23.51 | 662.0 | 20.49 |
| 19-072 | SQL-019 | 2019/7/16 13:45 | 98.8362 | 38.8336 | 3865 | 20.43 | 637.1 | 20.43 |
| 19-073 | SQL-020 | 2019/7/16 14:30 | 98.7453 | 38.7842 | 4138 | 18.02 | 616.3 | 20.37 |
| 19-074 | SQL-021 | 2019/7/16 15:18 | 98.4150 | 38.8134 | 3361 | 27.28 | 674.9 | 20.41 |
| 19-075 | SQL-022 | 2019/7/16 16:45 | 98.2795 | 39.0411 | 4303 | 18.60 | 603.5 | 20.42 |
| 19-076 | SQL-023 | 2019/7/16 17:33 | 98.2503 | 39.0939 | 3561 | 17.98 | 659.8 | 20.42 |
| 19-077 | SQL-024 | 2019/7/16 18:10 | 98.0556 | 39.1780 | 2998 | 22.25 | 704.9 | 20.46 |
| 19-078 | SQL-025 | 2019/7/16 18:50 | 97.9255 | 39.3110 | 2714 | 21.88 | 729.6 | 20.52 |
| 19-079 | SQL-026 | 2019/7/16 19:28 | 97.7082 | 39.3411 | 3609 | 14.50 | 656.6 | 20.42 |
| 19-080 | SQL-027 | 2019/7/16 19:50 | 97.6492 | 39.3673 | 4047 | 8.80 | 623.1 | 20.28 |
| 19-081 | SQL-028 | 2019/7/16 20:25 | 97.6864 | 39.5242 | 3161 | 15.12 | 693.2 | 20.29 |
| 19-082 | SQL-029 | 2019/7/17 8:28 | 98.2693 | 39.7990 | 1617 | 19.77 | 828.5 | 20.58 |
| 19-083 | SQL-030 | 2019/7/17 9:48 | 99.0575 | 39.3697 | 1569 | 23.84 | 837.1 | 20.55 |
| 19-084 | SQL-031 | 2019/7/17 10:50 | 99.4699 | 39.2240 | 1759 | 26.63 | 820.0 | 20.62 |
| 19-085 | SQL-032 | 2019/7/17 11:52 | 99.4697 | 39.0421 | 2491 | 19.34 | 754.9 | 20.61 |
| 19-086 | SQL-033 | 2019/7/17 12:30 | 99.5355 | 38.9327 | 2794 | 16.69 | 728.1 | 20.50 |
| 19-087 | SQL-034 | 2019/7/17 13:50 | 99.6586 | 38.8457 | 2234 | 21.48 | 775.6 | 20.48 |
| 19-088 | SQL-035 | 2019/7/17 14:15 | 99.8077 | 38.9238 | 2025 | 22.45 | 793.9 | 20.52 |
| 19-089 | SQL-036 | 2019/7/17 19:25 | 102.6323 | 37.9456 | 1497 | 29.85 | 840.5 | 20.71 |
| 19-090 | SQL-037 | 2019/7/18 9:35 | 102.7339 | 37.7337 | 1729 | 21.77 | 825.7 | 20.55 |
| 19-091 | SQL-038 | 2019/7/18 10:35 | 102.8738 | 37.5764 | 1909 | 23.50 | 808.5 | 20.56 |
| 19-092 | SQL-039 | 2019/7/18 11:18 | 102.8836 | 37.4234 | 2151 | 22.80 | 785.5 | 20.56 |
| 19-093 | SQL-040 | 2019/7/18 11:55 | 102.8942 | 37.2909 | 2485 | 20.75 | 755.5 | 20.53 |
| 19-094 | SQL-041 | 2019/7/18 12:35 | 102.8305 | 37.2094 | 2980 | 19.59 | 712.4 | 20.46 |
| 19-095 | SQL-042 | 2019/7/18 13:55 | 102.8651 | 37.1649 | 2800 | 17.98 | 726.9 | 20.37 |
| 19-096 | SQL-043 | 2019/7/18 14:35 | 103.0988 | 37.0141 | 2488 | 22.97 | 752.1 | 20.43 |
| 19-097 | SQL-044 | 2019/7/18 16:20 | 103.1579 | 36.9001 | 2322 | 22.33 | 767.8 | 20.49 |
| 19-098 | SQL-045 | 2019/7/18 17:35 | 103.2787 | 36.6974 | 2051 | 22.19 | 791.6 | 20.55 |
| 19-099 | SQL-046 | 2019/7/18 18:00 | 103.3790 | 36.5470 | 1879 | 24.85 | 807.3 | 20.59 |
| 19-100 | SQL-047 | 2019/7/18 19:35 | 103.8567 | 36.0485 | 1507 | 25.87 | 842.8 | 20.62 |
| 19-101 | SQL-048 | 2019/7/19 9:20 | 103.1643 | 36.1927 | 1648 | 20.30 | 833.3 | 20.48 |
| 19-102 | SQL-049 | 2019/7/19 9:45 | 102.9503 | 36.2942 | 1726 | 20.46 | 825.8 | 20.49 |
| 19-103 | SQL-050 | 2019/7/19 11:08 | 102.5681 | 36.4387 | 1897 | 24.45 | 809.6 | 20.53 |
| 19-104 | SQL-051 | 2019/7/19 11:40 | 102.4064 | 36.4852 | 2005 | 26.79 | 799.4 | 20.54 |
| 19-105 | SQL-052 | 2019/7/19 12:20 | 102.3966 | 36.6343 | 2410 | 23.09 | 762.7 | 20.55 |
| 19-106 | SQL-053 | 2019/7/19 13:00 | 102.4169 | 36.7154 | 2838 | 23.80 | 725.5 | 20.54 |
| 19-107 | SQL-054 | 2019/7/16 12:32 | 99.3110 | 38.6544 | 3474 | 25.33 | 667.1 | 20.51 |
| 19-108 | CZ-001 | 2019/7/29 8:50 | 91.1238 | 29.6510 | 3592 | 17.37 | 656.3 | 20.22 |
| 19-109 | CZ-002 | 2019/7/29 10:03 | 91.5063 | 29.7866 | 3723 | 14.66 | 650.1 | 20.25 |
| 19-110 | CZ-003 | 2019/7/29 10:40 | 91.6686 | 29.8087 | 3778 | 15.47 | 646.3 | 20.21 |
| 19-111 | CZ-004 | 2019/7/29 15:11 | 92.0700 | 29.7037 | 4136 | 24.28 | 616.2 | 20.33 |
| 19-112 | CZ-005 | 2019/7/29 15:57 | 92.3395 | 29.7879 | 4742 | 13.10 | 571.8 | 20.31 |
| 19-113 | CZ-006 | 2019/7/29 16:22 | 92.3450 | 29.8244 | 4990 | 16.02 | 555.4 | 20.17 |
| 19-114 | CZ-007 | 2019/7/29 16:51 | 92.4121 | 29.9103 | 4422 | 26.38 | 598.0 | 20.26 |
| 19-115 | CZ-008 | 2019/7/29 17:38 | 92.7935 | 29.9239 | 3919 | 21.66 | 634.5 | 20.41 |
| 19-116 | CZ-009 | 2019/7/29 18:33 | 93.2742 | 29.8810 | 3426 | 21.98 | 670.3 | 20.42 |
| 19-117 | CZ-010 | 2019/7/30 7:56 | 94.3706 | 29.6254 | 3040 | 15.82 | 710.5 | 20.29 |
| 19-118 | CZ-011 | 2019/7/30 8:41 | 94.5541 | 29.5590 | 3678 | 13.88 | 651.9 | 20.17 |
| 19-119 | CZ-012 | 2019/7/30 9:13 | 94.6522 | 29.6077 | 4501 | 8.66 | 589.2 | 20.06 |
| 19-120 | CZ-013 | 2019/7/30 9:29 | 94.6982 | 29.6135 | 4270 | 11.24 | 608.9 | 20.09 |
| 19-121 | CZ-014 | 2019/7/30 9:56 | 94.7296 | 29.6988 | 3472 | 15.45 | 673.4 | 20.19 |
| 19-122 | CZ-015 | 2019/7/30 10:32 | 94.7381 | 29.7657 | 3401 | 15.54 | 683.4 | 20.23 |
| 19-123 | CZ-016 | 2019/7/30 11:09 | 94.7999 | 29.9461 | 2572 | 17.93 | 750.8 | 20.31 |
| 19-124 | CZ-017 | 2019/7/30 12:00 | 95.0683 | 30.0960 | 2115 | 20.76 | 792.8 | 20.40 |
| 19-125 | CZ-018 | 2019/7/30 13:31 | 95.7394 | 29.8754 | 2708 | 20.56 | 733.9 | 20.41 |
| 19-126 | CZ-019 | 2019/7/30 15:45 | 96.0782 | 29.7416 | 3039 | 18.00 | 704.7 | 20.38 |
| 19-127 | CZ-020 | 2019/7/30 16:09 | 96.2062 | 29.6842 | 3128 | 18.03 | 697.1 | 20.34 |
| 19-128 | CZ-021 | 2019/7/30 20:20 | 96.6897 | 29.4894 | 3877 | 12.52 | 635.4 | 20.21 |
| 19-129 | CZ-022 | 2019/7/31 7:57 | 96.9167 | 30.0520 | 3265 | 16.00 | 687.4 | 20.26 |
| 19-130 | CZ-023 | 2019/7/31 8:24 | 97.0407 | 30.0161 | 3159 | 17.17 | 703.1 | 20.26 |
| 19-131 | CZ-024 | 2019/7/31 9:01 | 97.2365 | 30.0857 | 2717 | 19.96 | 734.3 | 20.30 |
| 19-132 | CZ-025 | 2019/7/31 9:35 | 97.3041 | 30.1102 | 3488 | 16.93 | 666.5 | 20.23 |
| 19-133 | CZ-026 | 2019/7/31 10:00 | 97.2946 | 30.1119 | 3920 | 14.88 | 633.3 | 20.18 |
| 19-134 | CZ-027 | 2019/7/31 10:26 | 97.2944 | 30.1474 | 4483 | 16.62 | 591.0 | 20.12 |
| 19-135 | CZ-028 | 2019/7/31 10:39 | 97.3107 | 30.1539 | 4604 | 12.65 | 583.3 | 20.13 |
| 19-136 | CZ-029 | 2019/7/31 11:03 | 97.2892 | 30.2046 | 4143 | 18.43 | 620.3 | 20.15 |
| 19-137 | CZ-030 | 2019/7/31 11:31 | 97.3211 | 30.1935 | 4106 | 19.24 | 622.4 | 20.19 |
| 19-138 | CZ-031 | 2019/7/31 15:29 | 97.6346 | 29.8906 | 3957 | 19.85 | 632.9 | 20.26 |
| 19-139 | CZ-032 | 2019/7/31 16:16 | 97.8481 | 29.6636 | 3799 | 17.09 | 644.9 | 20.28 |
| 19-140 | CZ-033 | 2019/7/31 16:48 | 97.9504 | 29.7298 | 4487 | 9.66 | 590.4 | 20.20 |
| 19-141 | CZ-034 | 2019/7/31 17:09 | 98.0021 | 29.7107 | 5026 | 3.60 | 551.4 | 20.06 |
| 19-142 | CZ-035 | 2019/8/1 7:54 | 98.2388 | 29.5373 | 3491 | 11.48 | 668.1 | 20.02 |
| 19-143 | CZ-036 | 2019/8/1 17:30 | 98.3497 | 29.6094 | 2845 | 26.06 | 729.6 | 20.34 |
| 19-144 | CZ-037 | 2019/8/1 18:13 | 98.3815 | 29.6839 | 3141 | 21.48 | 696.3 | 20.36 |
| 19-145 | CZ-038 | 2019/8/1 18:31 | 98.4416 | 29.7134 | 3648 | 18.01 | 652.2 | 20.30 |
| 19-146 | CZ-039 | 2019/8/1 18:53 | 98.4941 | 29.7024 | 4105 | 12.26 | 616.4 | 20.24 |
| 19-147 | CZ-040 | 2019/8/1 21:46 | 98.9490 | 29.7513 | 2614 | 20.44 | 745.0 | 20.45 |
| 19-148 | CZ-041 | 2019/8/2 7:28 | 99.1100 | 30.0058 | 2581 | 19.75 | 742.7 | 20.37 |
| 19-149 | CZ-042 | 2019/8/2 8:36 | 99.3949 | 30.2989 | 3478 | 14.89 | 662.4 | 20.24 |
| 19-150 | CZ-043 | 2019/8/2 9:04 | 99.4861 | 30.2820 | 4049 | 14.18 | 620.7 | 20.18 |
| 19-151 | CZ-044 | 2019/8/2 9:25 | 99.5730 | 30.2841 | 4621 | 9.65 | 578.5 | 20.04 |
| 19-152 | CZ-045 | 2019/8/2 9:48 | 99.7873 | 30.2176 | 4130 | 16.46 | 613.1 | 20.13 |
| 19-153 | CZ-046 | 2019/8/2 10:24 | 100.1028 | 30.0881 | 4004 | 21.76 | 625.7 | 20.17 |
| 19-154 | CZ-047 | 2019/8/2 11:17 | 100.4191 | 30.0782 | 4082 | 19.53 | 620.8 | 20.24 |
| 19-155 | CZ-048 | 2019/8/2 14:12 | 100.4826 | 30.0821 | 4387 | 16.59 | 597.3 | 20.16 |
| 19-156 | CZ-049 | 2019/8/2 14:51 | 100.6437 | 30.1427 | 4425 | 12.08 | 594.9 | 20.15 |
| 19-157 | CZ-050 | 2019/8/2 16:01 | 100.9037 | 29.9835 | 3653 | 13.14 | 656.8 | 20.26 |
| 19-158 | CZ-051 | 2019/8/2 16:29 | 100.9915 | 30.0258 | 2884 | 16.18 | 722.1 | 20.30 |
| 19-159 | CZ-052 | 2019/8/2 16:43 | 101.0262 | 30.0331 | 2669 | 17.24 | 740.9 | 20.34 |
| 19-160 | CZ-053 | 2019/8/2 17:22 | 101.2746 | 30.0388 | 3454 | 14.38 | 670.2 | 20.25 |
| 19-161 | CZ-054 | 2019/8/2 17:56 | 101.4028 | 30.0519 | 3834 | 13.06 | 638.5 | 20.21 |
| 19-162 | CZ-055 | 2019/8/3 8:02 | 101.9549 | 29.9958 | 2816 | 14.86 | 753.8 | 20.29 |
| 19-163 | CZ-056 | 2019/8/3 9:44 | 102.2378 | 29.9509 | 1626 | 25.83 | 837.7 | 20.43 |
| 19-164 | CZ-057 | 2019/8/3 10:35 | 102.7385 | 30.0297 | 907 | 29.47 | 908.9 | 20.60 |
| 19-165 | CZ-058 | 2019/8/3 11:48 | 103.1943 | 30.1606 | 743 | 31.99 | 922.0 | 20.69 |
| 19-166 | CZ-059 | 2019/8/3 12:43 | 103.4252 | 30.1827 | 645 | 29.42 | 932.5 | 20.78 |
| 20-001 | XK-001 | 2020/6/22 7:38 | 101.7362 | 36.6330 | 2277 | 14.56 | 771.8 | 20.30 |
| 20-002 | XK-002 | 2020/6/22 8:29 | 101.2835 | 36.6839 | 2596 | 15.35 | 741.1 | 20.35 |
| 20-003 | XK-003 | 2020/6/22 9:10 | 101.1010 | 36.4470 | 3416 | 11.86 | 670.4 | 20.26 |
| 20-004 | XK-004 | 2020/6/22 10:10 | 100.8449 | 36.3630 | 3208 | 14.24 | 690.6 | 20.23 |
| 20-005 | XK-005 | 2020/6/22 12:58 | 99.9902 | 35.8959 | 3250 | 16.15 | 686.5 | 20.37 |
| 20-006 | XK-006 | 2020/6/22 15:01 | 99.9043 | 35.8271 | 3914 | 7.09 | 632.7 | 20.18 |
| 20-007 | XK-007 | 2020/6/22 16:20 | 99.5115 | 35.4972 | 4486 | 5.68 | 589.7 | 20.18 |
| 20-008 | XK-008 | 2020/6/22 16:52 | 99.4363 | 35.4020 | 3960 | 13.99 | 631.3 | 20.11 |
| 20-009 | XK-009 | 2020/6/22 17:57 | 98.8654 | 35.1126 | 4239 | 11.36 | 609.1 | 20.11 |
| 20-010 | XK-010 | 2020/6/22 19:07 | 98.2143 | 34.8896 | 4237 | 13.75 | 609.3 | 20.11 |
| 20-011 | XK-011 | 2020/6/23 8:01 | 98.1229 | 34.7754 | 4343 | 7.10 | 602.9 | 20.12 |
| 20-012 | XK-012 | 2020/6/23 8:56 | 97.9768 | 34.4871 | 4332 | 5.42 | 605.0 | 20.11 |
| 20-013 | XK-013 | 2020/6/23 9:38 | 97.8906 | 34.2917 | 4597 | 3.83 | 585.2 | 20.05 |
| 20-014 | XK-014 | 2020/6/23 10:17 | 97.6578 | 34.1253 | 4817 | 3.19 | 569.5 | 20.00 |
| 20-015 | XK-015 | 2020/6/23 11:21 | 97.1439 | 33.7962 | 4424 | 9.40 | 598.8 | 20.10 |
| 20-016 | XK-016 | 2020/6/23 12:00 | 97.1899 | 33.6435 | 4413 | 14.28 | 599.4 | 20.12 |
| 20-017 | XK-017 | 2020/6/23 14:11 | 97.2967 | 33.4170 | 4278 | 14.19 | 609.5 | 20.13 |
| 20-018 | XK-018 | 2020/6/23 14:51 | 97.4870 | 33.2154 | 4376 | 13.54 | 601.2 | 20.19 |
| 20-019 | XK-019 | 2020/6/23 16:47 | 97.3562 | 33.1430 | 3926 | 21.86 | 637.2 | 20.36 |
| 20-020 | XK-020 | 2020/6/23 17:13 | 97.2500 | 33.0067 | 3596 | 21.99 | 662.6 | 20.40 |
| 20-021 | XK-021 | 2020/6/23 18:21 | 97.0078 | 32.9915 | 3731 | 20.51 | 650.5 | 20.32 |
| 20-022 | XK-022 | 2020/6/24 8:24 | 97.0399 | 32.8482 | 3890 | 9.51 | 641.5 | 20.09 |
| 20-023 | XK-023 | 2020/6/24 9:08 | 96.6945 | 32.8867 | 4470 | 10.59 | 594.0 | 20.09 |
| 20-024 | XK-024 | 2020/6/24 9:51 | 96.5954 | 32.6514 | 3945 | 12.34 | 635.7 | 20.20 |
| 20-025 | XK-025 | 2020/6/24 10:31 | 96.4581 | 32.5533 | 4309 | 14.38 | 605.7 | 20.15 |
| 20-026 | XK-026 | 2020/6/24 13:10 | 96.4883 | 32.2092 | 3654 | 23.22 | 656.8 | 20.26 |
| 20-027 | XK-027 | 2020/6/24 14:48 | 96.5106 | 31.9680 | 4290 | 19.14 | 604.3 | 20.22 |
| 20-028 | XK-028 | 2020/6/24 15:33 | 96.4148 | 31.9928 | 4123 | 21.77 | 618.9 | 20.28 |
| 20-029 | XK-029 | 2020/6/24 19:32 | 96.3198 | 31.8149 | 3686 | 21.88 | 653.0 | 20.37 |
| 20-030 | XK-030 | 2020/6/25 8:13 | 96.6061 | 31.2190 | 3829 | 11.09 | 642.4 | 20.10 |
| 20-031 | XK-031 | 2020/6/25 9:31 | 96.8636 | 31.1096 | 3900 | 14.46 | 635.1 | 20.05 |
| 20-032 | XK-032 | 2020/6/25 10:34 | 97.0514 | 31.1927 | 3338 | 18.10 | 680.1 | 20.29 |
| 20-033 | XK-033 | 2020/6/25 11:37 | 97.2919 | 31.0204 | 3195 | 24.50 | 688.8 | 20.31 |
| 20-034 | XK-034 | 2020/6/25 12:17 | 97.3455 | 30.8135 | 3134 | 28.89 | 692.5 | 20.38 |
| 20-035 | XK-035 | 2020/6/25 13:15 | 97.2658 | 30.6564 | 4371 | 19.16 | 592.9 | 20.27 |
| 20-036 | XK-036 | 2020/6/25 16:28 | 97.2164 | 30.4105 | 4235 | 24.26 | 606.7 | 20.18 |
| 20-037 | XK-037 | 2020/6/25 17:01 | 97.3213 | 30.1933 | 4111 | 17.84 | 615.9 | 20.25 |
| 20-038 | XK-038 | 2020/6/26 7:20 | 97.8457 | 29.6680 | 3800 | 15.71 | 640.7 | 20.26 |
| 20-039 | XK-039 | 2020/6/26 8:33 | 97.9642 | 29.7266 | 4662 | 9.92 | 574.3 | 20.03 |
| 20-040 | XK-040 | 2020/6/26 8:55 | 98.0021 | 29.7106 | 5048 | 7.90 | 548.0 | 19.99 |
| 20-041 | XK-041 | 2020/6/26 9:47 | 98.2296 | 29.5373 | 3528 | 21.44 | 665.8 | 20.19 |
| 20-042 | XK-042 | 2020/6/26 10:21 | 98.3167 | 29.5499 | 3907 | 17.28 | 632.2 | 20.21 |
| 20-043 | XK-043 | 2020/6/26 11:17 | 98.3543 | 29.6222 | 2718 | 29.72 | 730.9 | 20.40 |
| 20-044 | XK-044 | 2020/6/26 12:19 | 98.5245 | 29.6933 | 4319 | 20.59 | 597.7 | 20.31 |
| 20-045 | XK-045 | 2020/6/26 12:54 | 98.5974 | 29.6851 | 3881 | 24.15 | 634.3 | 20.32 |
| 20-046 | XK-046 | 2020/6/26 14:52 | 98.6743 | 29.3210 | 3534 | 27.94 | 660.8 | 20.37 |
| 20-047 | XK-047 | 2020/6/26 18:01 | 98.6808 | 29.2633 | 4182 | 12.73 | 608.4 | 20.24 |
| 20-048 | XK-048 | 2020/6/26 19:18 | 98.6309 | 28.9676 | 2328 | 26.71 | 764.7 | 20.45 |
| 20-049 | XK-049 | 2020/6/27 7:17 | 98.9177 | 28.4797 | 3200 | 14.66 | 689.4 | 20.18 |
| 20-050 | XK-050 | 2020/6/27 9:07 | 99.0969 | 28.3268 | 3989 | 13.24 | 626.2 | 20.10 |
| 20-051 | XK-051 | 2020/6/27 10:03 | 99.2751 | 28.2542 | 2576 | 24.72 | 746.2 | 20.35 |
| 20-052 | XK-052 | 2020/6/27 10:38 | 99.3951 | 28.1567 | 2051 | 29.38 | 791.7 | 20.50 |
| 20-053 | XK-053 | 2020/6/27 11:35 | 99.6031 | 27.9374 | 3462 | 17.45 | 665.1 | 20.36 |
| 20-054 | XK-054 | 2020/6/27 13:27 | 99.7093 | 27.8352 | 3286 | 24.40 | 683.7 | 20.33 |
| 20-055 | XK-055 | 2020/6/27 14:21 | 99.8103 | 27.4731 | 3222 | 21.49 | 689.0 | 20.37 |
| 20-056 | XK-056 | 2020/6/27 15:13 | 99.9879 | 27.3267 | 2387 | 27.15 | 763.3 | 20.47 |
| 20-057 | XK-057 | 2020/6/27 16:12 | 100.0739 | 27.0011 | 1869 | 26.40 | 807.6 | 20.53 |
| 20-058 | XK-058 | 2020/6/28 7:16 | 100.2266 | 26.8727 | 2390 | 20.45 | 758.7 | 20.53 |
| 20-059 | XK-059 | 2020/6/28 8:49 | 100.1315 | 26.8274 | 2488 | 21.58 | 753.2 | 20.41 |
| 20-060 | XK-060 | 2020/6/28 9:53 | 99.9685 | 26.6814 | 2274 | 24.94 | 772.2 | 20.46 |
| 20-061 | XK-061 | 2020/6/28 10:51 | 99.9652 | 26.4245 | 2434 | 24.25 | 756.4 | 20.51 |
| 20-062 | XK-062 | 2020/6/28 11:21 | 99.9864 | 26.3285 | 2276 | 27.50 | 771.8 | 20.51 |
| 20-063 | XK-063 | 2020/6/28 12:14 | 100.0720 | 26.0179 | 1979 | 26.16 | 798.8 | 20.57 |
| 20-064 | XK-064 | 2020/6/28 14:13 | 100.1545 | 25.6958 | 2037 | 31.27 | 792.0 | 20.61 |
| 20-065 | XK-065 | 2020/6/28 15:24 | 100.3307 | 25.5106 | 2034 | 31.26 | 791.5 | 20.56 |
| 20-066 | XK-066 | 2020/6/28 16:11 | 100.4379 | 25.4361 | 1790 | 31.81 | 815.6 | 20.66 |
| 20-067 | XK-067 | 2020/6/28 17:05 | 100.6086 | 25.4282 | 1991 | 29.78 | 795.1 | 20.64 |
| 20-068 | XK-068 | 2020/6/28 17:56 | 100.8391 | 25.3849 | 2217 | 27.07 | 773.3 | 20.59 |
| 20-069 | XK-069 | 2020/6/28 18:34 | 100.9809 | 25.2994 | 2438 | 25.33 | 753.3 | 20.54 |
| 20-070 | XK-070 | 2020/6/29 7:23 | 101.5387 | 25.0454 | 1784 | 22.98 | 817.8 | 20.43 |
| 20-071 | XK-071 | 2020/6/29 8:45 | 101.8806 | 24.9497 | 1549 | 25.98 | 841.2 | 20.50 |
| 20-072 | XK-072 | 2020/6/29 9:29 | 102.2589 | 24.9722 | 1833 | 25.85 | 812.2 | 20.52 |
| 20-073 | XK-073 | 2020/6/29 17:44 | 102.7065 | 25.0474 | 1809 | 27.40 | 803.5 | 20.48 |
| 20-074 | XK-074 | 2020/6/30 10:52 | 102.8881 | 24.9289 | 2044 | 27.59 | 791.5 | 20.47 |
| 20-075 | XK-075 | 2020/6/30 11:46 | 103.3358 | 24.8350 | 1797 | 29.91 | 816.6 | 20.56 |
| 20-076 | YA-001 | 2020/7/24 7:28 | 101.7504 | 36.6379 | 2278 | 18.05 | 775.4 | 20.20 |
| 20-077 | YA-002 | 2020/7/25 8:05 | 97.0078 | 32.9915 | 3731 | 14.29 | 655.8 | 20.24 |
| 20-078 | YA-003 | 2020/7/25 8:59 | 97.0373 | 32.8486 | 3864 | 17.46 | 642.8 | 20.14 |
| 20-079 | YA-004 | 2020/7/25 9:52 | 96.6945 | 32.8867 | 4457 | 12.82 | 596.4 | 20.12 |
| 20-080 | YA-005 | 2020/7/25 10:44 | 96.4050 | 32.9530 | 4244 | 16.98 | 614.2 | 20.17 |
| 20-081 | YA-006 | 2020/7/25 13:07 | 96.1018 | 32.9543 | 4238 | 18.94 | 614.9 | 20.24 |
| 20-082 | YA-007 | 2020/7/25 13:45 | 95.7553 | 32.9266 | 4720 | 17.82 | 577.2 | 20.18 |
| 20-083 | YA-008 | 2020/7/25 16:06 | 95.2472 | 32.8938 | 4156 | 24.60 | 619.5 | 20.38 |
| 20-084 | YA-009 | 2020/7/25 18:39 | 95.2534 | 32.8970 | 4112 | 23.42 | 622.0 | 20.37 |
| 20-085 | YA-010 | 2020/7/26 6:30 | 95.3037 | 32.8933 | 4005 | 5.85 | 628.3 | 20.10 |
| 20-086 | YA-011 | 2020/7/26 7:36 | 95.1497 | 32.7974 | 4703 | 8.49 | 581.0 | 19.97 |
| 20-087 | YA-012 | 2020/7/26 8:39 | 94.9995 | 32.8694 | 4313 | 8.57 | 610.8 | 20.14 |
| 20-088 | YA-013 | 2020/7/26 9:29 | 94.7710 | 32.9012 | 4664 | 8.77 | 583.7 | 20.16 |
| 20-089 | YA-014 | 2020/7/26 10:43 | 94.4266 | 32.8188 | 4707 | 15.32 | 581.1 | 20.20 |
| 20-090 | YA-015 | 2020/7/26 11:33 | 94.2911 | 32.8810 | 4882 | 15.92 | 568.6 | 20.21 |
| 20-091 | YA-016 | 2020/7/26 12:26 | 94.1774 | 32.9586 | 4773 | 16.91 | 576.9 | 20.24 |
| 20-092 | YA-017 | 2020/7/26 14:16 | 93.9746 | 33.0391 | 4907 | 15.68 | 566.7 | 20.26 |
| 20-093 | YA-018 | 2020/7/26 15:53 | 93.6575 | 33.0456 | 4720 | 19.00 | 580.1 | 20.28 |
| 20-094 | YA-019 | 2020/7/26 17:29 | 93.3841 | 32.7053 | 4823 | 15.83 | 571.6 | 20.29 |
| 20-095 | YA-020 | 2020/7/26 18:14 | 93.3009 | 32.6096 | 4944 | 14.32 | 562.7 | 20.21 |
| 20-096 | YA-021 | 2020/7/26 19:42 | 93.1238 | 32.3914 | 4497 | 16.27 | 595.8 | 20.31 |
| 20-097 | YA-022 | 2020/7/26 22:18 | 92.3066 | 32.1030 | 4635 | 12.78 | 583.7 | 20.31 |
| 20-098 | YA-023 | 2020/7/27 7:54 | 92.0614 | 31.4690 | 4491 | 9.53 | 591.8 | 20.04 |
| 20-099 | YA-024 | 2020/7/27 9:46 | 91.7320 | 31.6189 | 4547 | 17.43 | 589.1 | 20.05 |
| 20-100 | YA-025 | 2020/7/27 10:28 | 91.3938 | 31.5299 | 4603 | 19.93 | 584.7 | 20.14 |
| 20-101 | YA-026 | 2020/7/27 12:32 | 90.9308 | 31.3948 | 4550 | 20.01 | 588.6 | 20.21 |
| 20-102 | YA-027 | 2020/7/27 13:20 | 90.4145 | 31.3660 | 4620 | 25.40 | 582.9 | 20.24 |
| 20-103 | YA-028 | 2020/7/27 14:06 | 90.0136 | 31.3998 | 4713 | 22.07 | 576.2 | 20.28 |
| 20-104 | YA-029 | 2020/7/27 15:41 | 89.6697 | 31.5757 | 4608 | 27.95 | 583.4 | 20.24 |
| 20-105 | YA-030 | 2020/7/27 17:38 | 89.1504 | 31.5074 | 4610 | 18.56 | 582.0 | 20.22 |
| 20-106 | YA-031 | 2020/7/27 18:28 | 88.8042 | 31.6877 | 4588 | 9.26 | 584.4 | 20.23 |
| 20-107 | YA-032 | 2020/7/27 19:44 | 88.0955 | 31.8475 | 4758 | 15.21 | 571.0 | 20.16 |
| 20-108 | YA-033 | 2020/7/27 21:28 | 87.2389 | 31.7895 | 4540 | 16.60 | 587.5 | 20.17 |
| 20-109 | YA-034 | 2020/7/28 9:14 | 86.9068 | 31.9398 | 4504 | 12.15 | 590.7 | 20.08 |
| 20-110 | YA-035 | 2020/7/28 9:55 | 86.5139 | 31.9272 | 4703 | 10.55 | 576.0 | 20.05 |
| 20-111 | YA-036 | 2020/7/28 10:38 | 86.0537 | 31.8755 | 4792 | 12.79 | 570.2 | 20.06 |
| 20-112 | YA-037 | 2020/7/28 11:21 | 85.7658 | 31.9575 | 4993 | 12.92 | 556.5 | 20.09 |
| 20-113 | YA-038 | 2020/7/28 13:44 | 85.4001 | 32.0104 | 4848 | 17.18 | 563.6 | 20.07 |
| 20-114 | YA-039 | 2020/7/28 14:23 | 85.1591 | 31.9787 | 4566 | 21.42 | 586.0 | 20.16 |
| 20-115 | YA-040 | 2020/7/28 15:25 | 84.7945 | 32.0970 | 4446 | 22.49 | 593.6 | 20.24 |
| 20-116 | YA-041 | 2020/7/28 17:07 | 84.4283 | 32.2280 | 4506 | 22.74 | 587.7 | 20.18 |
| 20-117 | YA-042 | 2020/7/28 17:51 | 84.0646 | 32.3014 | 4443 | 24.90 | 592.4 | 20.27 |
| 20-118 | YA-043 | 2020/7/29 8:30 | 83.7086 | 32.3535 | 4366 | 11.45 | 600.2 | 20.06 |
| 20-119 | YA-044 | 2020/7/29 9:15 | 83.2160 | 32.4313 | 4448 | 15.32 | 594.6 | 20.08 |
| 20-120 | YA-045 | 2020/7/29 10:00 | 82.7863 | 32.4347 | 4478 | 13.40 | 592.5 | 20.12 |
| 20-121 | YA-046 | 2020/7/29 10:45 | 82.4486 | 32.3764 | 4867 | 10.94 | 564.2 | 20.10 |
| 20-122 | YA-047 | 2020/7/29 11:25 | 82.1354 | 32.1750 | 4833 | 19.86 | 566.4 | 20.10 |
| 20-123 | YA-048 | 2020/7/29 13:44 | 81.7251 | 32.1127 | 4748 | 22.40 | 573.9 | 20.12 |
| 20-124 | YA-049 | 2020/7/29 14:26 | 81.2529 | 32.2001 | 4612 | 27.11 | 582.9 | 20.21 |
| 20-125 | YA-050 | 2020/7/29 14:56 | 81.1360 | 32.3908 | 4539 | 28.09 | 587.6 | 20.28 |
| 20-126 | YA-051 | 2020/7/29 15:45 | 80.9291 | 32.4545 | 4496 | 27.64 | 589.9 | 20.29 |
| 20-127 | YA-052 | 2020/7/29 17:50 | 80.4816 | 32.3506 | 4638 | 23.00 | 578.1 | 20.32 |
| 20-128 | YA-053 | 2020/7/29 19:06 | 80.1023 | 32.5059 | 4313 | 26.51 | 602.1 | 20.34 |
| 20-129 | YA-054 | 2020/7/30 10:03 | 80.0830 | 32.1193 | 4293 | 16.98 | 607.9 | 20.16 |
| 20-130 | YA-055 | 2020/7/30 10:38 | 80.1065 | 31.9071 | 4532 | 20.74 | 589.1 | 20.15 |
| 20-131 | YA-056 | 2020/7/30 11:08 | 80.0725 | 31.8691 | 5280 | 14.86 | 535.8 | 20.07 |
| 20-132 | YA-057 | 2020/7/30 11:52 | 79.9362 | 31.8259 | 5362 | 13.36 | 531.9 | 20.08 |
| 20-133 | YA-058 | 2020/7/30 12:20 | 79.8333 | 31.8556 | 4693 | 17.25 | 580.8 | 20.18 |
| 20-134 | YA-059 | 2020/7/30 12:56 | 79.7480 | 31.6662 | 4074 | 23.68 | 627.2 | 20.30 |
| 20-135 | YA-060 | 2020/7/30 15:16 | 79.6722 | 31.4674 | 3751 | 31.53 | 648.0 | 20.44 |
| 20-136 | YA-061 | 2020/7/30 17:32 | 79.8052 | 31.4778 | 3755 | 29.43 | 643.7 | 20.43 |
| 20-137 | XC-001 | 2020/8/2 7:17 | 101.7355 | 36.6327 | 2277 | 18.10 | 772.1 | 20.39 |
| 20-138 | XC-002 | 2020/8/2 8:14 | 102.0219 | 36.4162 | 2414 | 20.03 | 759.1 | 20.39 |
| 20-139 | XC-003 | 2020/8/2 8:48 | 102.0029 | 36.1941 | 2636 | 18.91 | 739.5 | 20.43 |
| 20-140 | XC-004 | 2020/8/2 9:30 | 102.0022 | 36.0378 | 2126 | 23.98 | 787.2 | 20.51 |
| 20-141 | XC-005 | 2020/8/2 10:19 | 102.0722 | 35.6801 | 2270 | 26.42 | 772.8 | 20.55 |
| 20-142 | XC-006 | 2020/8/2 11:17 | 102.2592 | 35.5067 | 3278 | 21.31 | 683.3 | 20.45 |
| 20-143 | XC-007 | 2020/8/2 12:56 | 102.3302 | 35.4724 | 3623 | 19.99 | 656.8 | 20.36 |
| 20-144 | XC-008 | 2020/8/2 13:49 | 102.5165 | 35.2616 | 3372 | 25.69 | 677.3 | 20.41 |
| 20-145 | XC-009 | 2020/8/2 14:30 | 102.5362 | 35.2051 | 2948 | 25.64 | 714.3 | 20.52 |
| 20-146 | XC-010 | 2020/8/2 16:08 | 102.8184 | 35.2188 | 2560 | 34.02 | 746.1 | 20.55 |
| 20-147 | XC-011 | 2020/8/2 16:53 | 102.9103 | 34.9675 | 2911 | 29.20 | 714.0 | 20.57 |
| 20-148 | XC-012 | 2020/8/3 8:37 | 102.8350 | 34.8616 | 3015 | 17.40 | 706.8 | 20.22 |
| 20-149 | XC-013 | 2020/8/3 9:12 | 102.5791 | 34.7549 | 3198 | 19.07 | 690.7 | 20.27 |
| 20-150 | XC-014 | 2020/8/3 9:42 | 102.4677 | 34.5795 | 3146 | 19.16 | 695.6 | 20.31 |
| 20-151 | XC-015 | 2020/8/3 10:10 | 102.3699 | 34.5054 | 3437 | 18.06 | 671.0 | 20.31 |
| 20-152 | XC-016 | 2020/8/3 11:55 | 102.3057 | 34.3501 | 3586 | 20.73 | 659.6 | 20.26 |
| 20-153 | XC-017 | 2020/8/3 13:01 | 102.6411 | 34.0930 | 3342 | 25.86 | 680.6 | 20.35 |
| 20-154 | XC-018 | 2020/8/3 14:20 | 102.7538 | 33.9972 | 3472 | 26.38 | 666.8 | 20.40 |
| 20-155 | XC-019 | 2020/8/3 14:52 | 102.9393 | 33.8411 | 3467 | 26.94 | 667.6 | 20.40 |
| 20-156 | XC-020 | 2020/8/3 16:41 | 102.9328 | 33.5884 | 3457 | 25.38 | 667.7 | 20.42 |
| 20-157 | XC-021 | 2020/8/3 17:37 | 102.5522 | 33.4118 | 3610 | 20.68 | 654.6 | 20.42 |
| 20-158 | XC-022 | 2020/8/3 18:16 | 102.6161 | 33.1529 | 3465 | 20.90 | 666.5 | 20.40 |
| 20-159 | XC-023 | 2020/8/3 19:22 | 102.5394 | 32.7924 | 3502 | 21.57 | 663.4 | 20.41 |
| 20-160 | XC-024 | 2020/8/4 8:28 | 102.3284 | 32.6282 | 3509 | 13.91 | 664.0 | 20.16 |
| 20-161 | XC-025 | 2020/8/4 9:10 | 102.4469 | 32.3360 | 3807 | 18.84 | 639.9 | 20.20 |
| 20-162 | XC-026 | 2020/8/4 9:43 | 102.5174 | 32.1455 | 3486 | 19.63 | 667.2 | 20.32 |
| 20-163 | XC-027 | 2020/8/4 10:35 | 102.6328 | 31.8715 | 3176 | 27.42 | 693.9 | 20.36 |
| 20-164 | XC-028 | 2020/8/4 11:57 | 102.8893 | 31.5313 | 2608 | 25.11 | 744.3 | 20.47 |
| 20-165 | XC-029 | 2020/8/4 12:40 | 103.1141 | 31.4061 | 2020 | 31.91 | 797.2 | 20.56 |
| 20-166 | XC-030 | 2020/8/4 13:08 | 103.1685 | 31.4357 | 1933 | 34.34 | 803.8 | 20.60 |
| 20-167 | XC-031 | 2020/8/4 14:34 | 103.3399 | 31.5701 | 1619 | 31.71 | 831.4 | 20.67 |
| 20-168 | XC-032 | 2020/8/4 15:11 | 103.5685 | 31.5062 | 1394 | 35.64 | 852.5 | 20.68 |
| 20-169 | XC-033 | 2020/8/4 15:23 | 103.5786 | 31.4764 | 1368 | 34.88 | 854.4 | 20.70 |
| 20-170 | XC-034 | 2020/8/4 15:51 | 103.4960 | 31.3586 | 1243 | 30.77 | 867.0 | 20.73 |
| 20-171 | XC-035 | 2020/8/4 16:32 | 103.4872 | 31.1117 | 981 | 29.20 | 894.0 | 20.68 |
| 20-172 | XC-036 | 2020/8/4 16:59 | 103.4885 | 31.0548 | 897 | 30.48 | 902.3 | 20.71 |
| 20-173 | XC-037 | 2020/8/4 17:35 | 103.5001 | 31.0016 | 935 | 27.60 | 897.6 | 20.69 |
| 20-174 | XC-038 | 2020/8/4 18:35 | 103.6136 | 30.9498 | 716 | 28.79 | 920.5 | 20.69 |
| 20-175 | XC-039 | 2020/8/5 11:10 | 103.6074 | 31.0051 | 741 | 25.17 | 920.1 | 20.55 |
| 20-176 | XC-040 | 2020/8/5 17:34 | 104.0639 | 30.6475 | 534 | 33.01 | 943.2 | 20.61 |

Notes:

1. ID_year was the number of samples for each year. The ID was the number of samples in each field investigation.

2. Time: The Beijing Time when the sample was taken.

3. Latitude and Longitude: Latitude and longitude of the sample site, measured with a Garmin 63sc GPS, resolution: 1″ (the samples in 2017 were measured with a Garmin Oregon 450 GPS, resolution: 1″).

4. Altitude: The elevation (m above sea level) of the sample site, measured with a Garmin 63sc GPS, resolution: 1 m (the samples in 2017 were measured with a Garmin Oregon 450 GPS, resolution: 1 m).

5. Air temperature, barometric pressure were measured with a DPH-103 smart digital temperature-humidity-barometer, with resolutions of 0.01℃ and 0.1hPa, respectively (in 2017, air temperature was not measured, and barometric pressure was measured with a Casio prg 130gc Barometer, resolution: 5 hPa). Air temperature, barometric pressure and oxygen concentration were each measured simultaneously by three separate instruments. The above table shows the average.

6. Oxygen concentration was measured with a TD400-SH-O_2_ portable oxygen analyzer (resolution: 0.01% in 2018-2020; 0.1% in 2017, with a CY-12C digital oxygen analyzer).

**Table S2. Statistics of investigated oxygen concentration data**（2017-2020）

| **No.** | **Investigated Area** | **Time** | **Sample Size** | **Range (%)** | **Mean (%)** | **SD (%)** |
| --- | --- | --- | --- | --- | --- | --- |
| 1 | Lhasa-Nagqu-Golmud | 2017.07.27-08.02 | 65 | 20.80-21.90 | 21.10 | 0.20 |
| 2 | Lhasa-Ngari-Yecheng | 2018.08.01-08.10 | 67 | 19.94-20.66 | 20.15 | 0.14 |
| 3 | Qinghai Lake and its vicinities | 2018.08.15-08.17 | 13 | 20.28-20.59 | 20.40 | 0.09 |
| 4 | Qilian Mountains (Winter) | 2019.02.13-02.19 | 53 | 19.98-20.43 | 20.16 | 0.11 |
| 5 | Qilian Mountains (Summer) | 2019.07.14-07.20 | 54 | 20.09-20.71 | 20.47 | 0.10 |
| 6 | Lhasa-Nyingchi-Chengdu | 2019.07.27-08.04 | 59 | 20.02-20.78 | 20.27 | 0.14 |
| 7 | Xining-Yushu-Kunming | 2020.06.22-06.29 | 75 | 19.99-20.66 | 20.31 | 0.17 |
| 8 | Yushu-Nagqu-Ngari | 2020.07.24-07.30 | 61 | 19.97-20.44 | 20.19 | 0.10 |
| 9 | Xining-Hezuo-Chengdu | 2020.08.02-08.05 | 40 | 20.26-20.73 | 20.47 | 0.15 |
| Total | | | 487 | 19.94-20.78 | 20.28 | 0.18 |

**Table S3. Oxygen concentrations sampled around Qilian mountains during summer and winter, 2019**

| No. | Altitude (m) | Summer (July) | | Winter (Feb.) | |
| --- | --- | --- | --- | --- | --- |
|  |  | Air temperature (℃) | Oxygen concentration (%) | Air temperature (℃) | Oxygen concentration (%) |
| 1 | 2346 | 16.44 | 20.42 | -4.37 | 20.30 |
| 2 | 2588 | 18.88 | 20.42 | -2.50 | 20.15 |
| 3 | 3377 | 15.24 | 20.44 | -9.10 | 20.13 |
| 4 | 3102 | 18.84 | 20.37 | -1.85 | 20.05 |
| 5 | 2379 | 20.95 | 20.52 | -0.83 | 20.16 |
| 6 | 2611 | 23.80 | 20.56 | 3.71 | 20.20 |
| 7 | 3013 | 20.36 | 20.50 | 8.31 | 20.26 |
| 8 | 3254 | 13.42 | 20.54 | 3.07 | 20.26 |
| 9 | 3770 | 11.95 | 20.35 | -3.36 | 20.18 |
| 10 | 3546 | 17.05 | 20.34 | -2.86 | 20.03 |
| 11 | 3130 | 16.09 | 20.46 | 3.36 | 20.09 |
| 12 | 2860 | 15.96 | 20.45 | -0.50 | 20.22 |
| 13 | 2735 | 11.25 | 20.28 | 0.89 | 20.19 |
| 14 | 4150 | 11.17 | 20.09 | -11.58 | 19.94 |
| 15 | 2693 | 15.97 | 20.42 | -7.32 | 20.06 |
| 16 | 3075 | 23.28 | 20.41 | -4.58 | 20.07 |
| 17 | 3292 | 25.12 | 20.50 | -2.64 | 20.07 |
| 18 | 3552 | 23.51 | 20.49 | -0.05 | 20.05 |
| 19 | 3865 | 20.43 | 20.43 | -0.73 | 20.10 |
| 20 | 4138 | 18.02 | 20.37 | -6.42 | 19.98 |
| 21 | 3361 | 27.28 | 20.41 | 1.93 | 20.02 |
| 22 | 4303 | 18.60 | 20.42 | -2.29 | 20.06 |
| 23 | 3561 | 17.98 | 20.42 | 2.96 | 20.20 |
| 24 | 2998 | 22.25 | 20.46 | 5.36 | 20.23 |
| 25 | 2714 | 21.88 | 20.52 | 1.63 | 20.29 |
| 26 | 3609 | 14.50 | 20.42 | -1.76 | 20.16 |
| 27 | 4047 | 8.80 | 20.28 | -7.87 | 20.06 |
| 28 | 3161 | 15.12 | 20.29 | -7.16 | 20.05 |
| 29 | 1617 | 19.77 | 20.58 | -11.54 | 20.43 |
| 30 | 1569 | 23.84 | 20.55 | -8.69 | 20.18 |
| 31 | 1759 | 26.63 | 20.62 | -6.86 | 20.20 |
| 32 | 2491 | 19.34 | 20.61 | -3.21 | 20.04 |
| 33 | 2794 | 16.69 | 20.50 | -1.09 | 20.07 |
| 34 | 2234 | 21.48 | 20.48 | 1.10 | 20.18 |
| 35 | 2025 | 22.45 | 20.52 | -2.12 | 20.24 |
| 36 | 1497 | 29.85 | 20.71 | -1.19 | 20.40 |
| 37 | 1729 | 21.77 | 20.55 | -5.32 | 20.25 |
| 38 | 1909 | 23.50 | 20.56 | -3.92 | 20.17 |
| 39 | 2151 | 22.80 | 20.56 | -6.55 | 20.09 |
| 40 | 2485 | 20.75 | 20.53 | -4.71 | 20.10 |
| 41 | 2980 | 19.59 | 20.46 | -7.35 | 20.03 |
| 42 | 2800 | 17.98 | 20.37 | -3.98 | 20.00 |
| 43 | 2488 | 22.97 | 20.43 | -0.88 | 20.14 |
| 44 | 2322 | 22.33 | 20.49 | -0.79 | 20.15 |
| 45 | 2051 | 22.19 | 20.55 | 1.22 | 20.22 |
| 46 | 1879 | 24.85 | 20.59 | 2.02 | 20.27 |
| 47 | 1507 | 25.87 | 20.62 | 2.32 | 20.39 |
| 48 | 1648 | 20.30 | 20.48 | -1.54 | 20.27 |
| 49 | 1726 | 20.46 | 20.49 | -0.25 | 20.25 |
| 50 | 1897 | 24.45 | 20.53 | 0.74 | 20.31 |
| 51 | 2005 | 26.79 | 20.54 | 1.41 | 20.24 |
| 52 | 2410 | 23.09 | 20.55 | 1.97 | 20.18 |
| 53 | 2838 | 23.80 | 20.54 | 1.77 | 20.13 |

**Table S4. Monthly mean and SD of oxygen concentration at Fangshan Station from Mar. 2019 to Feb. 2020**

| Year | Month | Mean (%) | SD (%) |
| --- | --- | --- | --- |
| 2019 | 3 | 20.84 | 0.29 |
| 2019 | 4 | 21.05 | 0.18 |
| 2019 | 5 | 21.24 | 0.19 |
| 2019 | 6 | 21.31 | 0.14 |
| 2019 | 7 | 21.28 | 0.13 |
| 2019 | 8 | 21.21 | 0.14 |
| 2019 | 9 | 21.12 | 0.17 |
| 2019 | 10 | 20.88 | 0.21 |
| 2019 | 11 | 20.62 | 0.20 |
| 2019 | 12 | 20.36 | 0.18 |
| 2020 | 1 | 20.29 | 0.20 |
| 2020 | 2 | 20.40 | 0.18 |

**Table S5. The FVCs of 33 quadrat samples and their counterparts retrieved from remote sensing data**

| ID_year | Altitude (m) | FVC_MODIS (%) | FVC_GLASS (%) | FVC_GE (%) | FVC (%) | LAI |
| --- | --- | --- | --- | --- | --- | --- |
| 18-059 | 3864 | 0.14 | 0.00 | 6.17 | 7.90 | 0.05 |
| 18-055 | 5010 | 0.00 | 0.00 | 11.11 | 10.20 | 0.17 |
| 18-047 | 4258 | 9.45 | 0.00 | 12.35 | 10.95 | 0.14 |
| 20-115 | 4446 | 3.27 | 0.00 | 17.28 | 11.85 | 0.07 |
| 20-126 | 4496 | 6.22 | 0.00 | 19.75 | 16.16 | 0.08 |
| 20-122 | 4833 | 8.61 | 0.00 | 20.99 | 17.67 | 0.16 |
| 18-075 | 3139 | 12.78 | 0.00 | 22.22 | 25.20 | 0.25 |
| 18-026 | 4586 | 30.44 | 4.40 | 23.46 | 25.90 | 0.11 |
| 18-065 | 2188 | 0.06 | 0.00 | 19.75 | 29.90 | 0.11 |
| 18-032 | 4635 | 30.26 | 6.20 | 22.22 | 30.20 | 0.13 |
| 18-014 | 3943 | 44.65 | 6.80 | 19.75 | 33.00 | 0.22 |
| 20-037 | 4111 | 59.08 | 19.20 | 43.21 | 34.91 | 2.41 |
| 20-112 | 4993 | 20.77 | 2.80 | 34.57 | 36.57 | 0.19 |
| 19-139 | 4106 | 57.01 | 10.60 | 44.44 | 43.20 | 1.95 |
| 20-104 | 4608 | 20.16 | 0.00 | 46.91 | 43.60 | 0.26 |
| 20-100 | 4603 | 55.50 | 12.40 | 61.73 | 46.20 | 0.71 |
| 20-016 | 4413 | 82.99 | 41.60 | 54.32 | 47.06 | 0.30 |
| 18-050 | 4265 | 14.55 | 0.00 | 6.17 | 51.80 | 0.09 |
| 18-018 | 5122 | 73.55 | 33.60 | 41.98 | 52.40 | 0.32 |
| 20-051 | 2576 | 90.30 | 30.80 | 58.02 | 53.46 | 1.98 |
| 19-112 | 3778 | 95.99 | 46.00 | 64.20 | 61.30 | 1.40 |
| 20-004 | 3208 | 97.11 | 57.20 | 82.72 | 61.44 | 1.98 |
| 20-035 | 4371 | 11.58 | 26.40 | 72.84 | 65.14 | 2.68 |
| 20-018 | 4376 | 100.00 | 64.40 | 76.54 | 70.29 | 0.80 |
| 20-080 | 4244 | 98.36 | 48.40 | 92.59 | 73.33 | 1.27 |
| 19-156 | 4082 | 100.00 | 78.80 | 65.43 | 75.40 | 3.14 |
| 20-028 | 4123 | 100.00 | 62.00 | 86.42 | 77.82 | 5.38 |
| 20-025 | 4309 | 100.00 | 61.60 | 86.42 | 78.16 | 0.79 |
| 20-083 | 4156 | 96.02 | 63.20 | 87.65 | 81.19 | 1.70 |
| 20-155 | 3467 | 100.00 | 84.40 | 85.19 | 87.52 | 3.91 |
| 18-070 | 3831 | 99.90 | 61.40 | 90.12 | 92.20 | 2.82 |
| 20-142 | 3278 | 100.00 | 77.20 | 70.37 | 92.95 | 3.38 |
| 20-151 | 3437 | 100.00 | 90.00 | 93.83 | 96.60 | 3.60 |

**Notes：**FVC_GE was calculated using Google Earth^18^. FVC_GLASS was downloaded from the Global Land Surface Satellite (GLASS) Product (250 m × 250 m, http://www.glass.umd.edu). FVC_MODIS was derived from Moderate Resolution Imaging Spectroradiometer (MODIS) MOD13Q1 products (https://ladsweb.modaps.eosdis.nasa.gov)^S1^. FVC was measured in the field with 1 km × 1 km survey sample area during 2018-2020^37^. LAI was downloaded and extracted from the Global Land Surface Satellite (GLASS) Product (0.05° × 0.05°, http://www.glass.umd.edu).

**
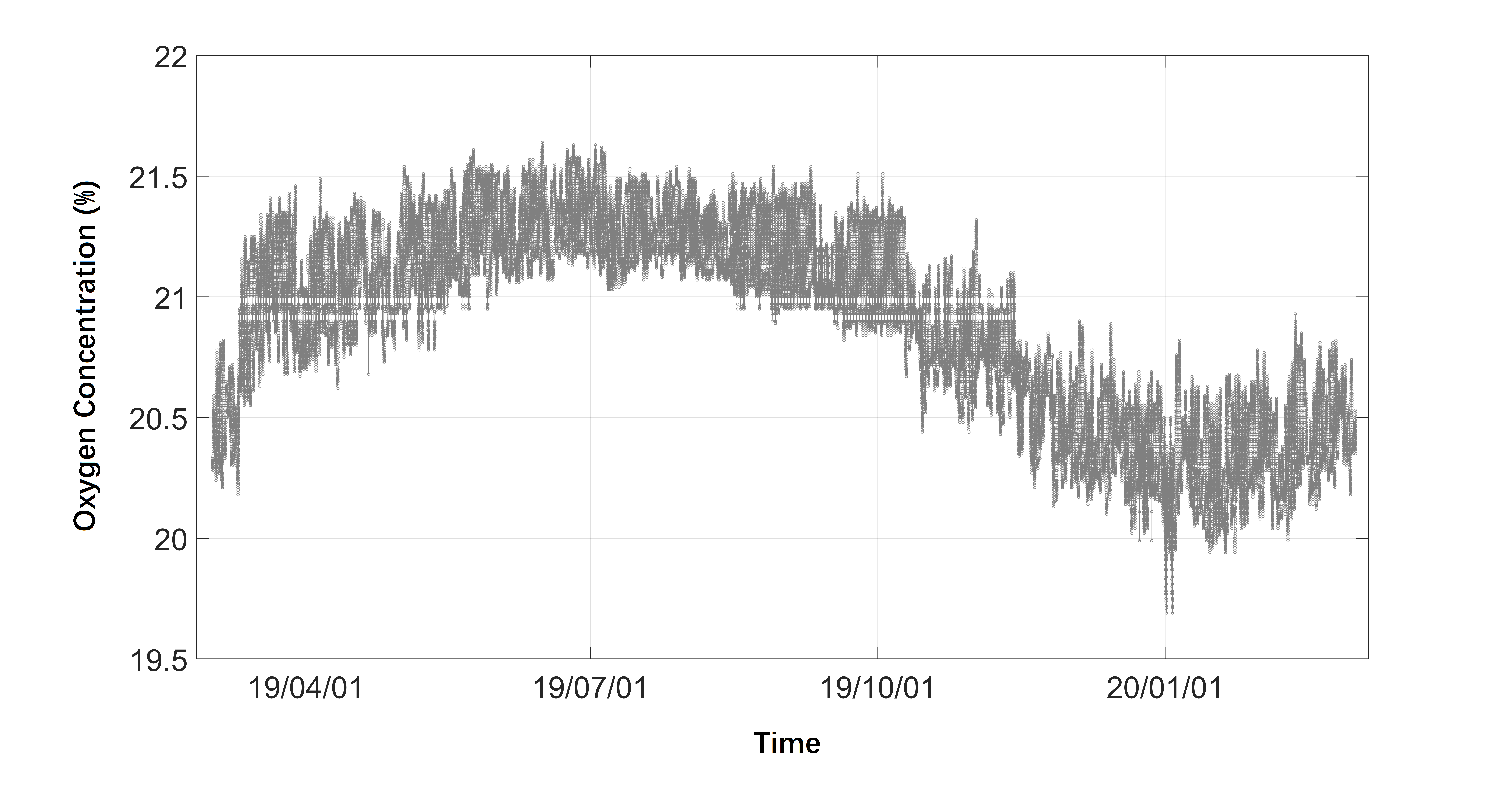
**

**Fig. S1 Oxygen concentration at Fangshan Station, Beijing from Mar. 2019 to Feb. 2020**

**
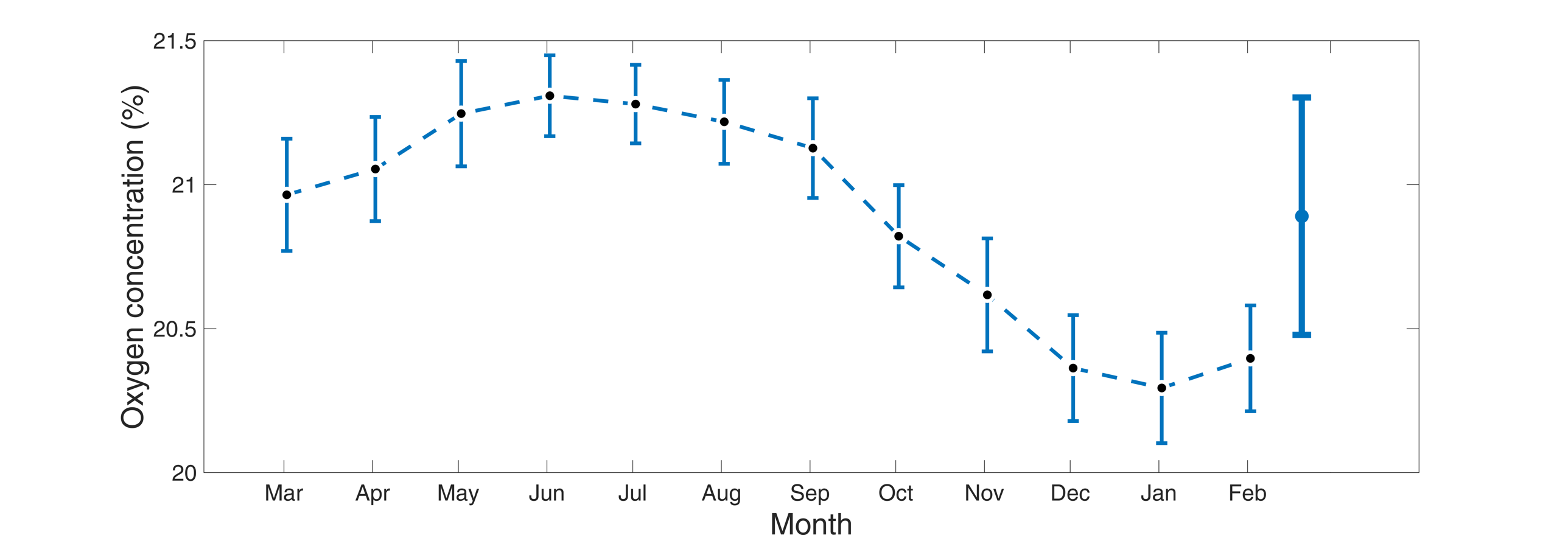
**

**Fig. S2 Monthly variations of oxygen concentration at Fangshan Station, Beijing from Mar. 2019 to Feb. 2020.** The left blank dots and blue bar represent the means and SDs of oxygen concentration in each month. The far-right blue dot and bar denote the mean and SD of oxygen concentration for the whole year.

**
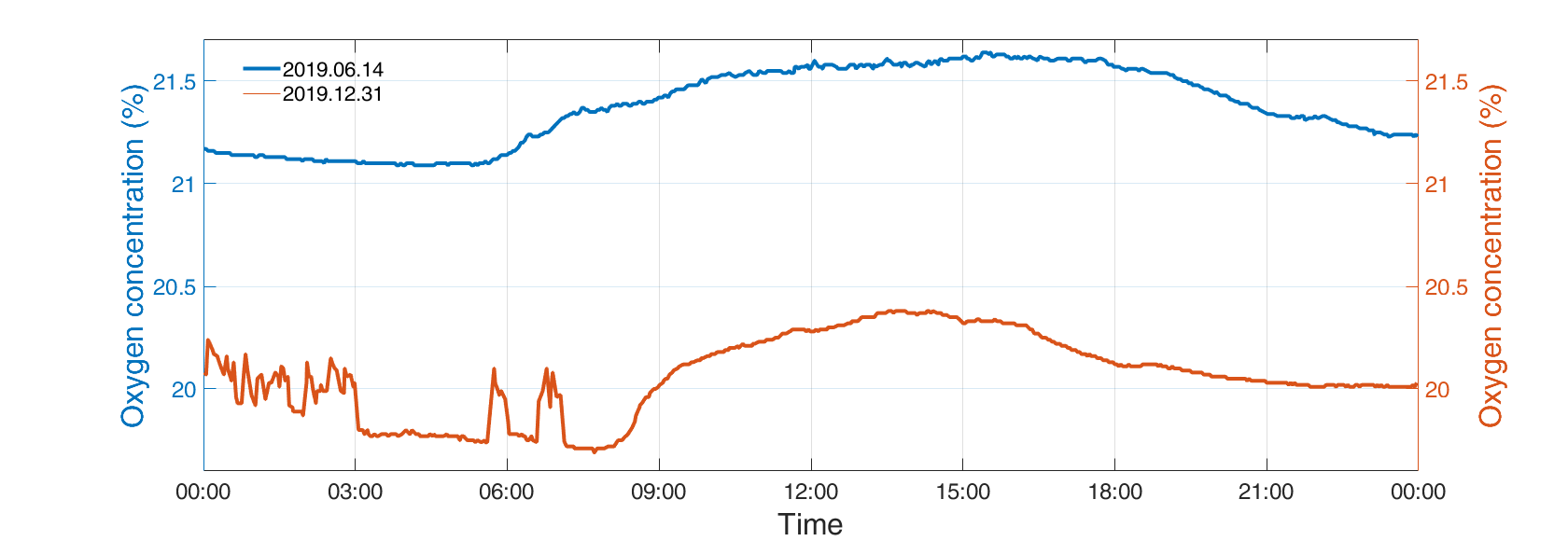
**

**Fig. S3 Diurnal curves of O_2_ concentration at Fangshan Station on June 14 and December 31, 2019**


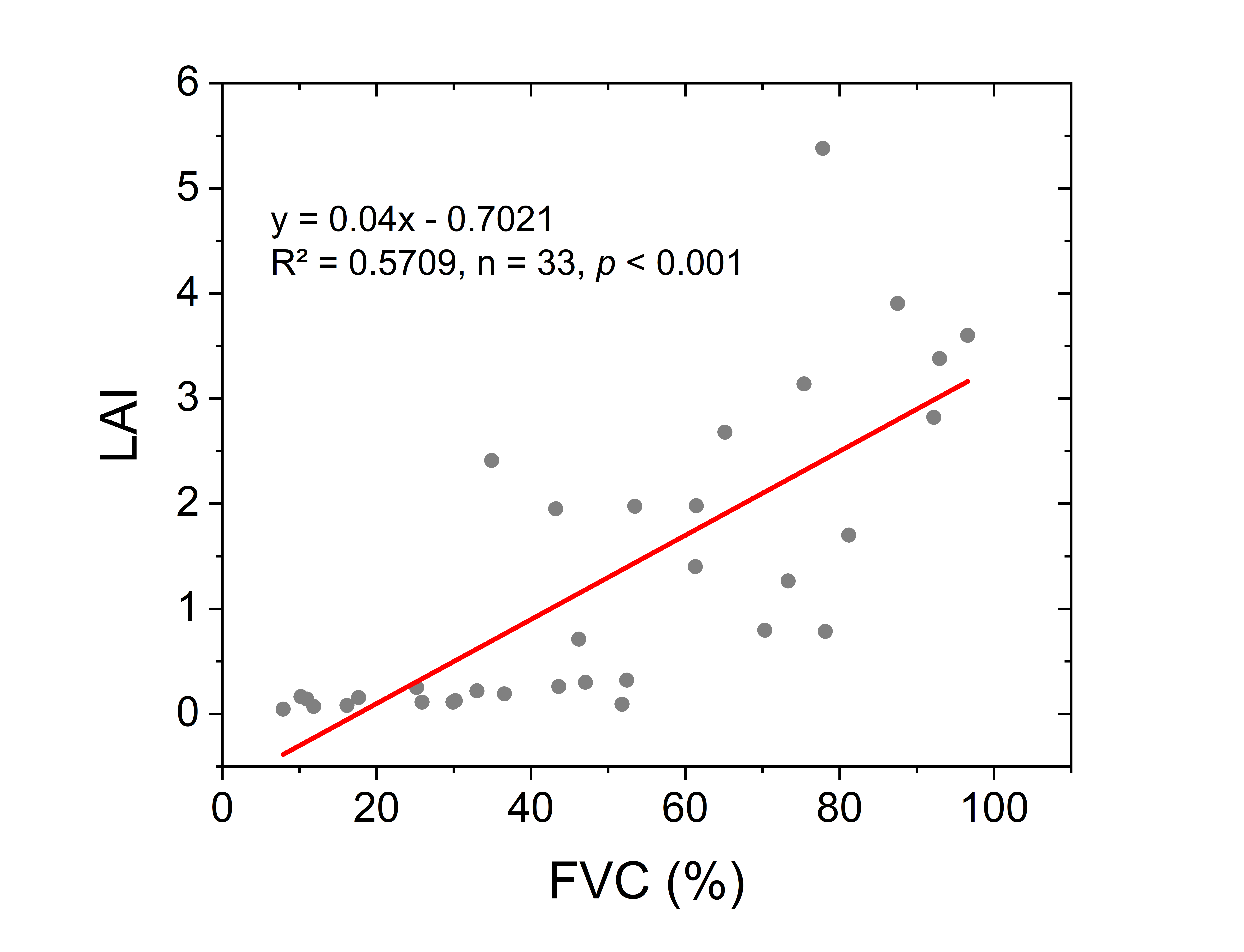

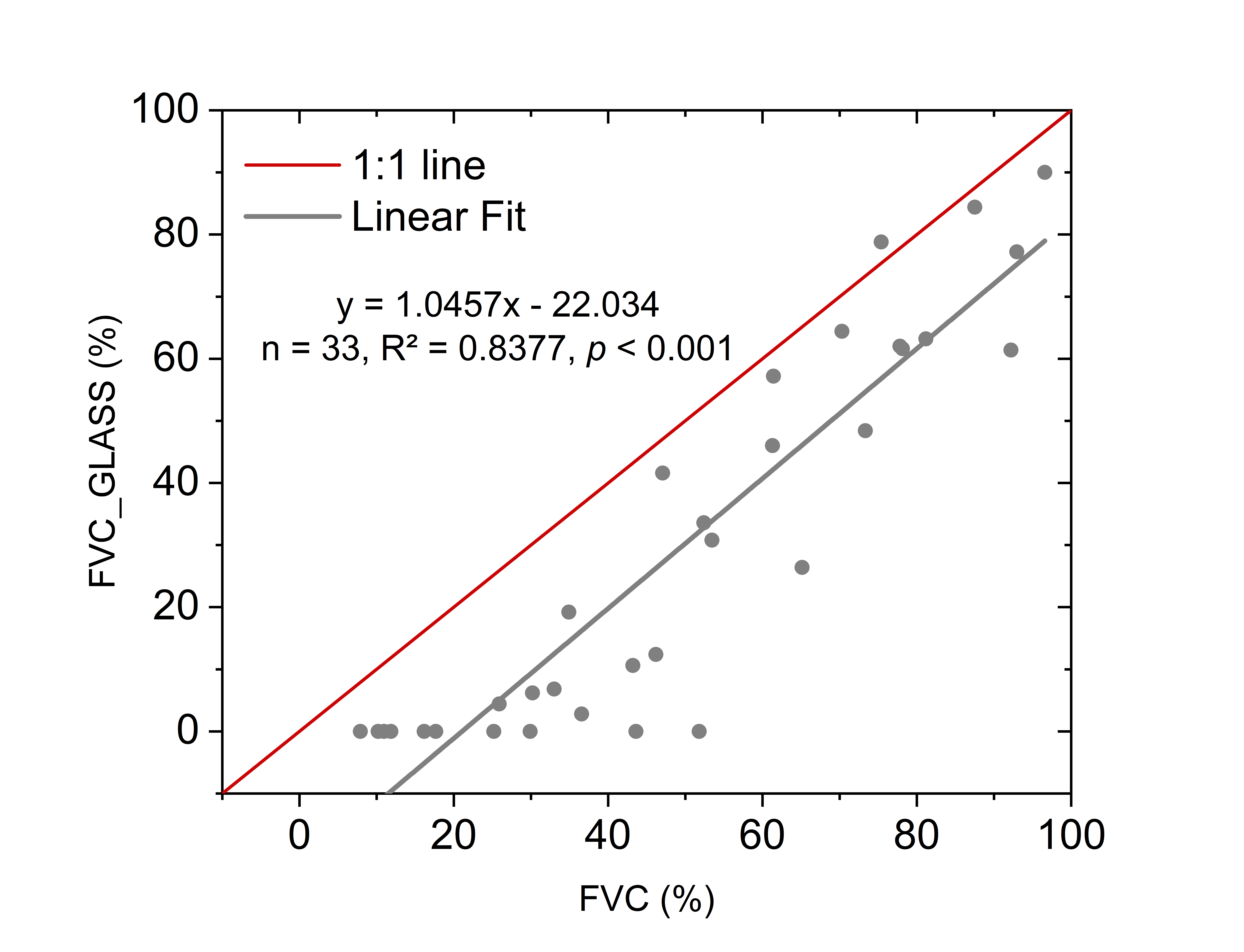


**Fig. S4 Scatter plots of FVC and LAI, FVC and FVC_GLASS.** The red line in the left represents the linearly fitted trend of FVC and LAI. The red line in the right represent the 1:1 line, and the gray one depicts the linearly fitted trend of FVC and FVC_GLASS.

**
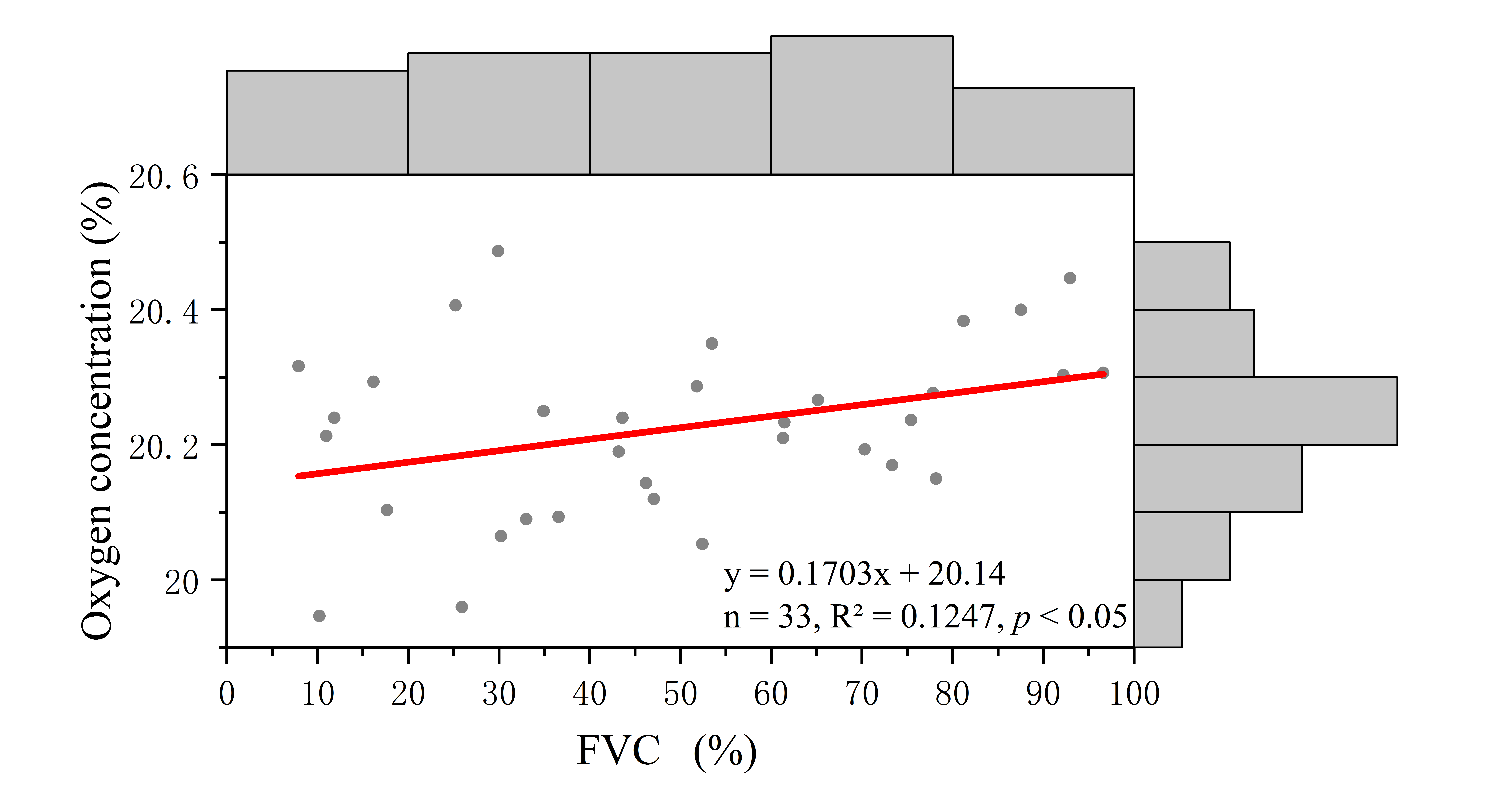
**
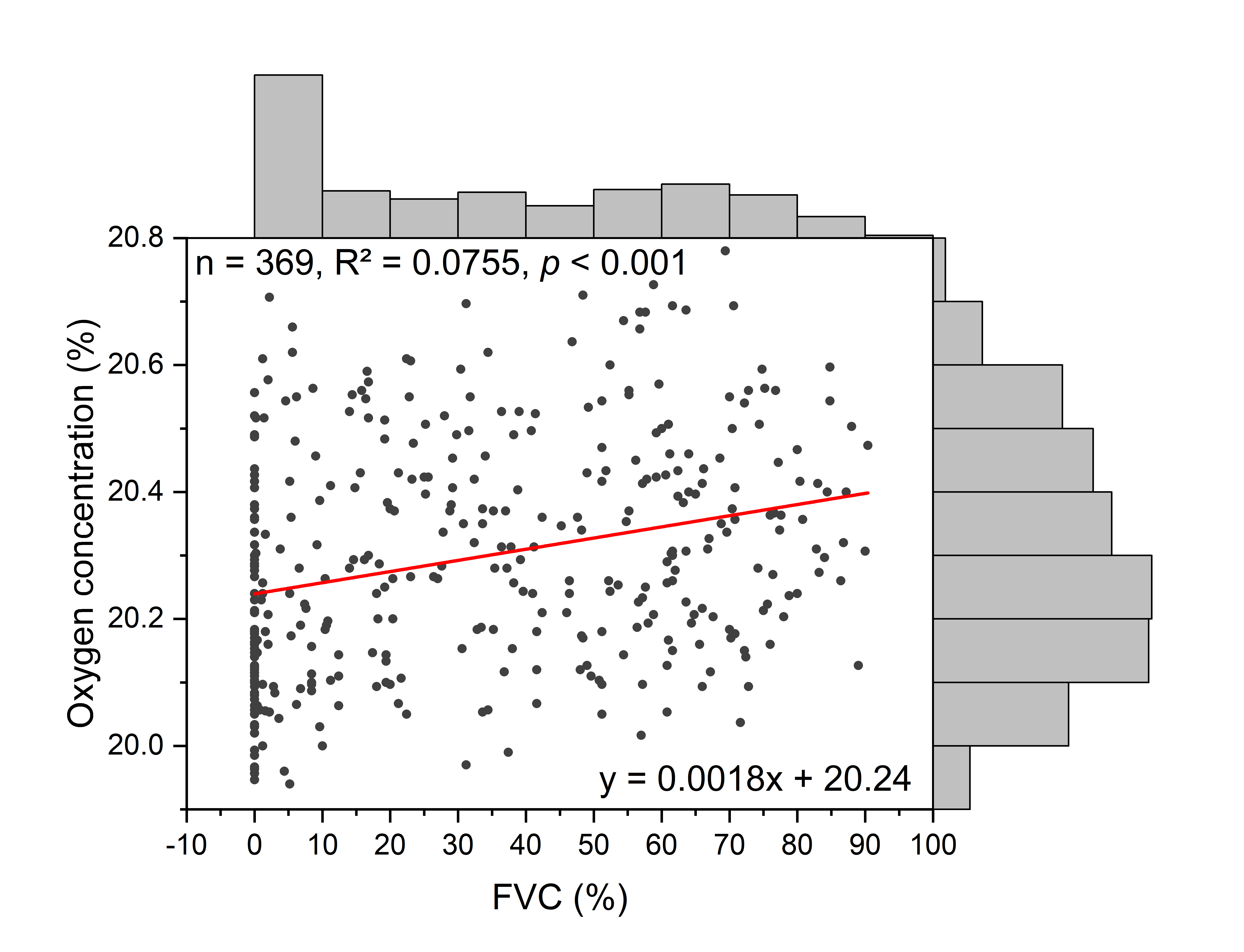


**Fig. S5 Scatter plots of oxygen concentration and FVC.** Left: Samples of which FVC were field-investigated, n =33; Right: All the samples except those in winter of 2019, n = 369. The red line indicates the linearly fitted trend of oxygen concentration and FVC. The upper and right histograms depict the frequency distribution of FVC and oxygen concentration, respectively.

**References**

1. Gutman G, Ignatov A. The derivation of the green vegetation fraction from NOAA/AVHRR data for use in numerical weather prediction models. International Journal of Remote Sensing, 1998, 19(8): 1533-1543.
